# Mechanistic insights into remodeled Tau-derived PHF6 peptide fibrils by Naphthoquinone-Tryptophan hybrids

**DOI:** 10.1038/s41598-017-18443-2

**Published:** 2018-01-08

**Authors:** V. Guru KrishnaKumar, Ashim Paul, Ehud Gazit, Daniel Segal

**Affiliations:** 1 Department of Molecular Microbiology and Biotechnology, Tel Aviv University, Ramat Aviv, Tel Aviv, 69978 Israel; 2 Department of Biological Engineering, Indian Institute of Technology, Gandhinagar, Palaj, Gandhinagar, Gujarat 382355 India; 3 Department of Materials Science and Engineering Iby and Aladar Fleischman Faculty of Engineering, Tel Aviv University, Ramat Aviv, Tel Aviv, 69978 Israel; 4 Interdisciplinary Sagol School of Neurosciences, Tel-Aviv University, Tel Aviv, 69978 Israel

## Abstract

Intra-cellular tau protein tangles and extra-cellular β-amyloid plaques are hallmarks of Alzheimer’s disease (AD), characterized by the conversion of natively unfolded monomeric protein/peptide into misfolded β-sheet rich aggregates. Therefore, inhibiting the aggregation cascade or disassembling the pre-formed aggregates becomes a pivotal event in disease treatment. In the present study, we show that Naphthoquinone-Tryptophan hybrids, i.e., NQTrp and Cl-NQTrp significantly disrupted the pre-formed fibrillar aggregates of Tau-derived PHF6 (VQIVYK) peptide and full-length tau protein *in vitro*, in a dose-dependent manner as evident from ThS assay, CD spectroscopy, and TEM. Molecular dynamics simulation of PHF6 oligomers and fibrils with the Naphthoquinone-Tryptophan hybrids provides a possible structure-function based mechanism-of-action, highlighting the role of hydrophobic interaction and hydrogen bond formation during fibril disassembly. These findings signify the effectiveness of NQTrp and Cl-NQTrp in disassembling fibrillar aggregates and may help in designing novel hybrid molecules for AD treatment.

## Introduction

Alzheimer’s disease (AD) is the primary cause of dementia and an archetypal example of proteinopathic disorders, in which aggregation of intra-neuronal tau protein and extracellular β-amyloid (Aβ) peptide acts as universal denominator in manifesting disease^1–3^. Amongst tau and Aβ, the role of tau in AD pathology has gained significant importance as the severity of AD is better correlated with the deposition of Neurofibrillary Tangles (NFTs) of the tau protein than senile plaques of Aβ^4^. Tau is a neuronal protein which promotes the assembly of tubulin monomers into microtubules (MT) in the axons and thus plays a key role in cytoskeleton stabilization^5,6^. Active functioning of the tau protein is regulated by controlled Post Translational Modifications (PTMs) such as phosphorylation and glycosylation, occurring at specific amino acid residues^7–9^. But in AD brain, the tau protein undergoes abnormal PTMs which promote the self-assembly of natively unfolded soluble monomers into insoluble amyloid aggregates such as Paired Helical Filaments (PHFs) and NFTs^10,11^. *In vivo* and *in vitro* assays performed on oligomers and higher order aggregates suggest that these assemblies consist of β-sheet rich fibrillar conformations, which are highly toxic to neuronal cells^10–15^. Under disease conditions, the formation of misfolded protein aggregates is a progressive process leading to increasing loads of amyloid deposits in AD brain^16^. The major challenge in AD and other related tauopathies including Parkinson’s disease (PD) and Huntington’s disease (HD) is the clearance of existing amyloid fibrils. The problem is multifaceted in the brain where the cellular regeneration and renewal mechanism is severely affected by age. Therefore, there is an immediate need for lead compounds which can arrest the amyloid aggregation as well as destabilize the pre-formed fibrils.

In spite of several small molecules and peptidomimetic compounds shown to inhibit the amyloid formation and disassemble pre-formed fibrils *in vitro*, currently, there are no prevailing disease-modifying therapies for amyloid-associated disorders. Natural products such as polyphenols, alkaloids, dyes, and synthetic biomaterials comprising of functionalized nanoparticles and peptides are widely studied for their anti-amyloidogenic behavior, and some were shown to alleviate toxicity of amyloids to living cells^17–20^. *Pickhardt et al*. had shown anthraquinones to inhibit tau protein aggregation and dissolve PHFs *in vitro* and in cells^21^. Also, *Paranjape et al*. have demonstrated that azaphilone derivatives inhibit and dissolve tau aggregates *in vitro* and found that specific arrangement of electron-withdrawing group could enhance tau fibril disassembly^22^. *Caruana et al*. found that polyphenols having a) aromatic elements and b) vicinal hydroxyl groups present on a single phenyl ring could effectively inhibit and destabilize α-synuclein fibrils^23^. *Du et al*. thoroughly studied the efficacy of brazilin in disrupting the intermolecular salt bridge between Asp^23^-Lys^28^ of amyloid β aggregates via hydrogen bonding resulting in disaggregation of pre-formed fibrils^24^. A recent study by *Ghosh et al*. showed the ability of an 11-mer peptide (NF11) to interact with the N-terminus region as well as the central hydrophobic cluster of Aβ40 to stop fibrillization and diminish existing mature fibrils^25^. Clearly, there is an interest to find potent molecules with “dual functionalities” i.e. (i) inhibition of aggregation (ii) disintegration of amyloids and to understand the mechanism by which they do so.

The initiation of tau protein aggregation is believed to be mediated by a hexapeptide core fragment ^306^VQIVYK^311^ (PHF6) located in the third repeat of the MT-binding region of the tau protein^26,27^. Supporting this statement, the recently reported cryo-EM structure of tau filaments demonstrated that the N-terminal part of the cross-β structure is formed by the PHF6 fragment and this hexapeptide is essential for the self-assembly propensity of tau^28^. Since the PHF6 peptide can form β-sheet rich conformation similar to tau-oligomers and NFTs; this hexapeptide is widely used as a model to screen and study potent anti-amyloidogenic compounds for therapeutics^8,29,30^. We have previously described the ability of Naphthoquinone-Tryptophan hybrids to inhibit the aggregation of PHF6 peptide *in vitro* and ameliorate the AD symptoms in transgenic Drosophila fly models expressing the human tau protein^31,32^. To the best of our knowledge, the disassembly of tau-based hexapeptide fibrils using NQTrp hybrids has not been examined previously. Since Naphthoquinone-Tryptophan hybrids are established generic inhibitors of amyloid aggregation^33^, we wished to determine whether they possess “dual functionality” towards mature PHF6 peptide fibrils. Using *in vitro* methods, we examined the ability of NQTrp and Cl-NQTrp to disrupt pre-formed fibrillar aggregates of PHF6 and full-length tau protein. Using *in silico* approaches, we demonstrate the putative interaction sites between peptides in the fibrillar strands and the hybrid molecules, which could suggest a possible mechanism of action adopted during fibril disassembly. The structure-function insights regarding the activity of Naphthoquinone-Tryptophan hybrids towards amyloidic aggregates may provide a route for designing targeted drugs for AD and other proteinopathies.

## Results and Discussion

**Figure 1:**
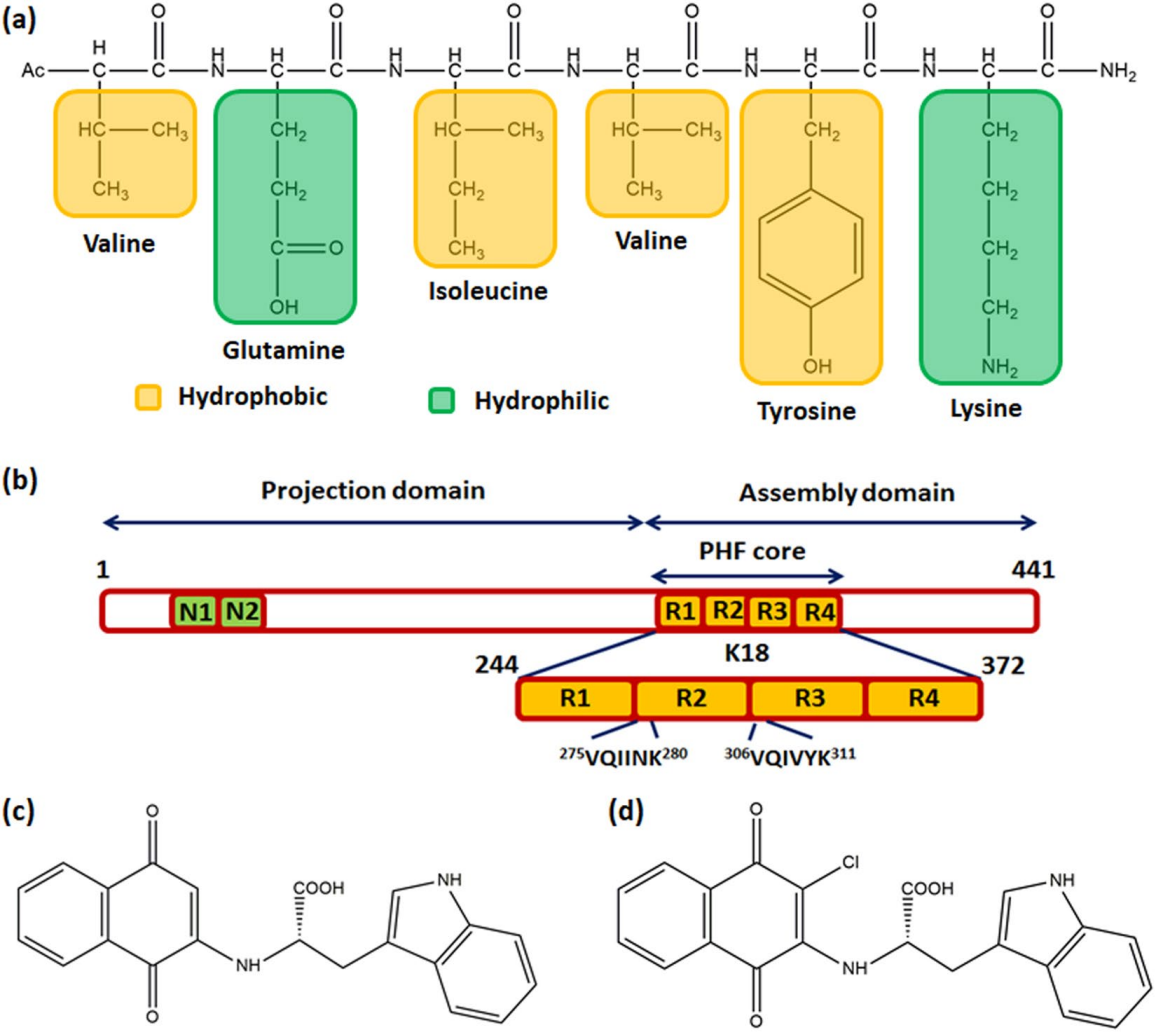
Molecular structures of (**a**) PHF6 hexapeptide (**b**) Full-length tau protein (**c**) NQTrp (**d**) Cl-NQTrp.

In the present work, we have used the PHF6 hexapeptide (VQIVYK, Fig. 1a) as a model system to understand the disassembly of pre-formed fibrils and from the insights obtained, we have extended our studies with full length tau protein (Fig. 1b) implicated in Alzheimer’s disease (AD). Since the AD-affected brain is rich in mature amyloid fibrils and highly toxic oligomers, the need of reducing existing amyloid load becomes crucial for treatment and drug design^34,35^. To that end, we have tested the efficacy of Naphthoquinone-Tryptophan hybrid molecules, i.e. NQTrp and Cl-NQTrp (Fig. 1c,d) on the pre-formed fibrillar assembly using various *in vitro* techniques and aimed at providing a plausible mechanism of disassembly by molecular dynamics simulation.

### NQTrp hybrids reduce amyloid load by fibril disassembly

**Figure 2:**
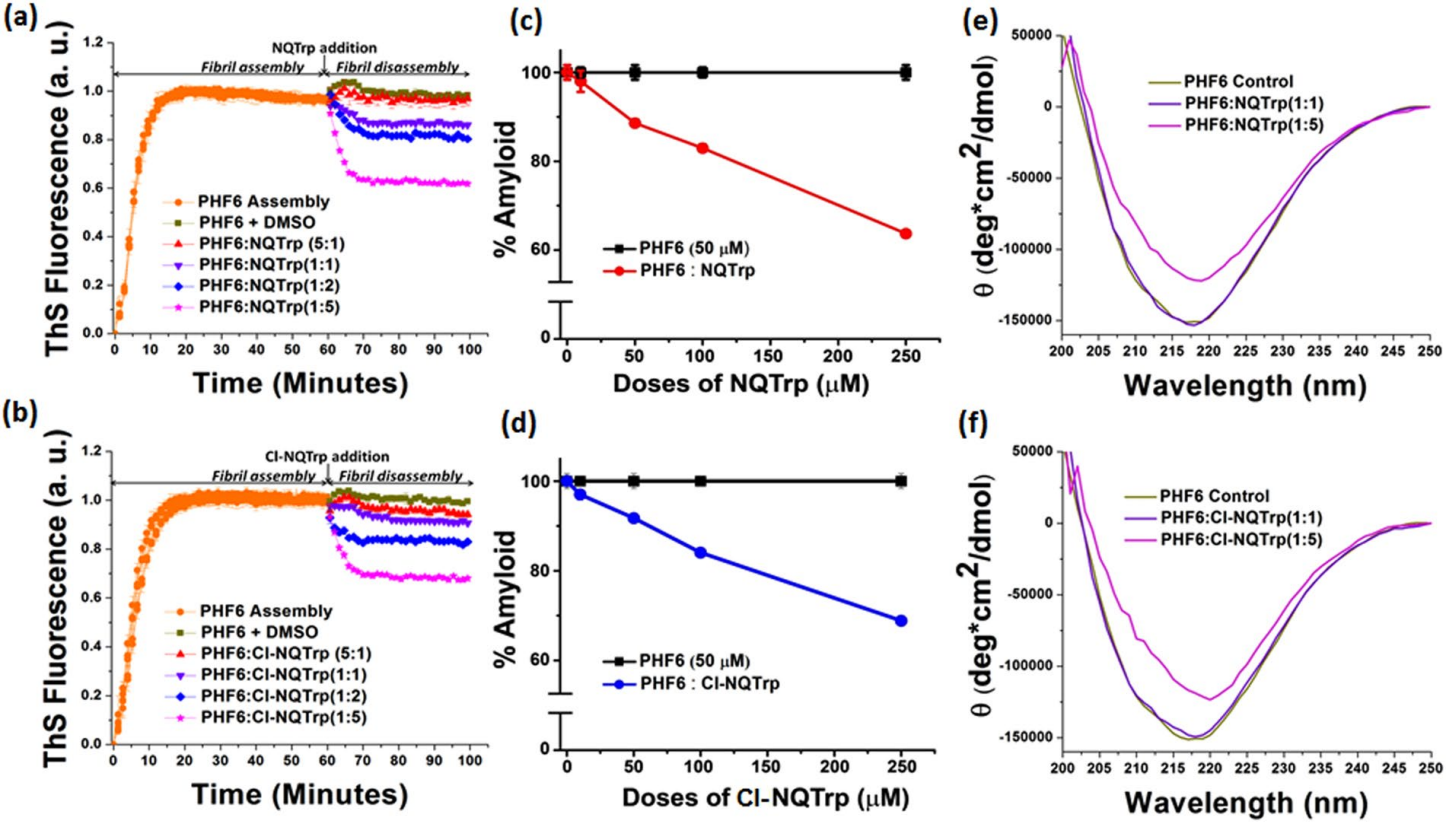
ThS fluorescence assay showing disassembly of pre-formed PHF6 peptide fibrils in presence of (**a**) NQTrp (**b**) Cl-NQTrp. Plot showing percentage amyloid remaining in the mixture after the fibril disassembly by (**c**) NQTrp (**d**) Cl-NQTrp. Circular dichroism spectroscopy analysis of disassembled PHF6 peptide fibrils in the presence of (**e**) NQTrp (**f**) Cl-NQTrp.

We designed an experiment in which PHF6 peptide was initially allowed to form amyloid fibrils, and then NQTrp or Cl-NQTrp were separately added to the pre-formed fibrils at different doses (5:1, 1:1, 1:2 and 1:5 PHF6: compound, respectively). Probable disassembly of fibrils, effected by the hybrid molecules was tested using a series of *in vitro* experiments including Thioflavin S (ThS) fluorescence assay, Transmission EM and CD spectroscopy. Thioflavin S assay indicated that 50 µM PHF6 peptide in presence of 10 µM heparin formed amyloid fibrils within 20 minutes. To obtain a clear demarcation between assembly and disassembly, we allowed the assembly reaction to progress until 60 minutes so that a smooth plateau of the ThS fluorescence curve was attained. At the end of 60 minutes, the hybrid molecules were added separately in 0.2-, 1-, 2-, and 5-fold molar excess into the pre-formed fibrillar assembly. Then, the mixture was co-incubated for additional 40 min, during which the kinetics of disassembly process was monitored by the fall in ThS fluorescence. As shown in Fig. 2a,b, the drop in ThS fluorescence became significant with increased doses of the hybrid molecules. Maximum disassembly of fibrils was observed in presence of 5-fold molar excess, followed by 2-fold excess. However, a 0.2-fold molar excess of the hybrid molecules was not sufficient for disrupting the pre-formed fibrils.

Since ThS is an amyloid reporter dye the fluorescence intensity could be a direct measure of amyloid content. To quantify the disassembly brought by the hybrid molecules, a plot of the percentage of amyloids vs. doses of hybrid molecules were generated. As shown in Fig. 2c,d both the hybrid molecules were equally efficient in significantly disassembling the pre-formed amyloids. The effect of hybrid molecules on the secondary structure changes of PHF6 fibrils was monitored by circular dichroism (CD) spectroscopy (Fig. 2e,f). The CD analysis indicated that PHF6 control fibrils, i.e., in the absence of hybrid molecules had β-sheet rich conformation. In contrast, the pre-formed fibrils co-incubated with the increased doses of the hybrid molecules, exhibited lower negative maxima at θ_218_, indicating a reduction in β-sheet content.

### Morphology of remodeled fibrils

**Figure 3:**
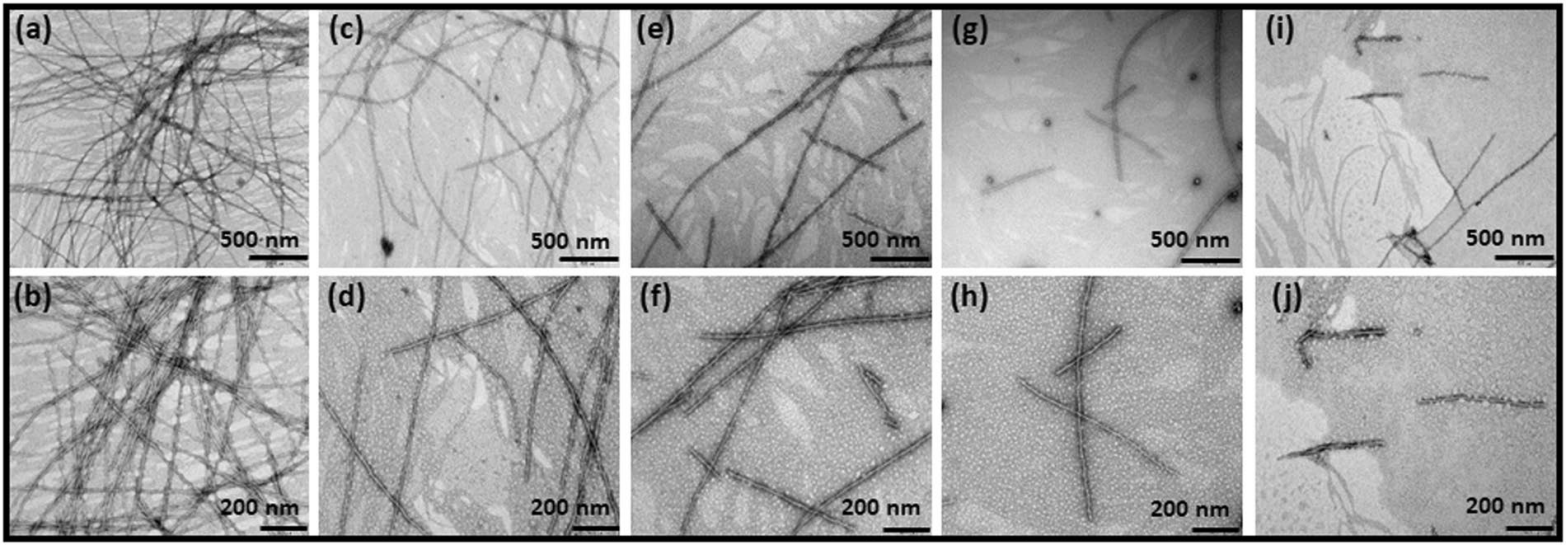
Representative TEM images showing disassembled PHF6 fibrils in the absence and presence of various molar ratio of PHF6:NQTrp/Cl-NQTrp (**a**,**b**) Control PHF6 fibrils treated with DMSO (**c**,**d**) NQTrp [1:1] (**e**,**f**) Cl-NQTrp [1:1] (**g**,**h**) NQTrp [1:5] (**i**,**j**) Cl-NQTrp [1:5].

**Figure 4:**
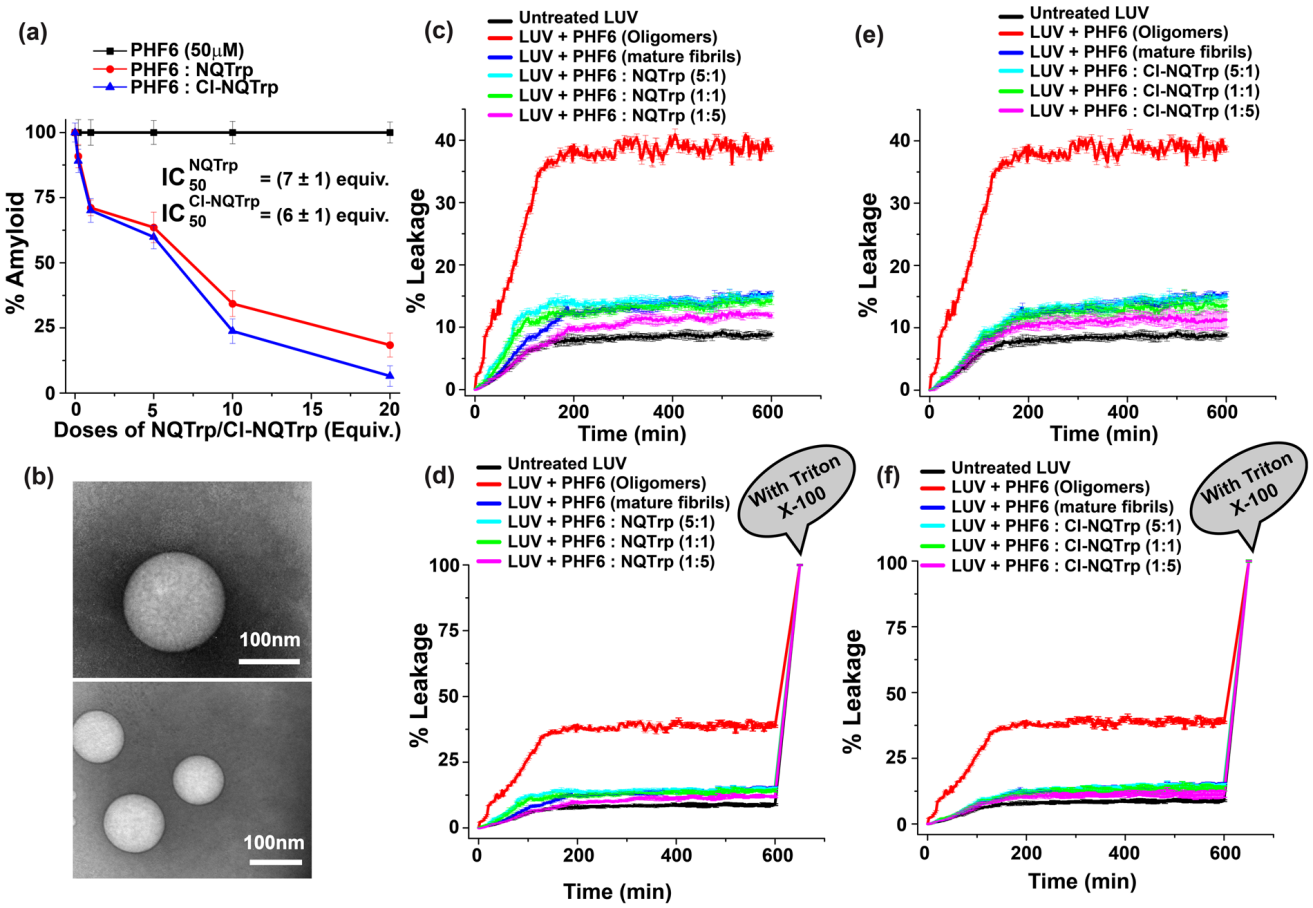
(**a**) IC_50_ values of the hybrid molecules for fibril disassembly was calculated using the ThS assay. (**b**) TEM images of the large unilamellar vesicles (LUVs) at concentration of 50 µM (stock concentration 1 mM) in MOPS buffer (20 mM). Images were taken immediately after LUVs preparation. The effect of PHF6 aggregation on LUVs were monitored by carboxyfluorescein dye emission and % of dye leakage. (**c**,**e**) Dye leakage from LUVs in absence and presence of different PHF6 preparations till 600 min. (**d**,**f**) Dye leakage from LUVs in absence and presence of different PHF6 preparations showing complete dye release after treatment with Triton X-100.

Transmission Electron Microscopy (TEM) analysis of the pre-formed PHF6 fibrils was performed following incubation treatment with NQTrp or Cl-NQTrp at molar ratio concentrations 1:1 and 1:5 (PHF6: NQTrp/Cl-NQTrp). Representative images of disassembled PHF6 fibrils are shown in Fig. 3. In the presence of DMSO (control) heparin-induced aggregation of PHF6 was mature and long (Fig. 3a,b). In contrast, shorter fibrils appeared with broken morphology following treatment with of 1:1 NQTrp or Cl-NQTrp (Fig. 3c–f). At 5-fold molar excess of the hybrid molecules, the density of the PHF6 fibrils was significantly reduced and no elongated fibrillar assemblies were observed, indicating a significant disassembly of fibrils (Fig. 3g–j). Next, we calculated the fibril density from the TEM images of individual samples (Supplementary Fig. 1). We observed that the average number of fibrils present per µm^2^ area of PHF6 control i.e., in the absence of hybrid molecules to be 77 ± 17. However, this value was reduced to 20 ± 07, 18 ± 04 in presence of PHF6:NQTrp (1:1), PHF6:Cl-NQTrp (1:1) and further to 07 ± 04, 08 ± 04 with PHF6:NQTrp (1:5), PHF6:Cl-NQTrp (1:5), respectively. These results support the fact that there prevailed a significant reduction in PHF6 fibril density after the treatment with hybrid molecules. The TEM data was also in agreement with the drop in amyloid content as determined by ThS assay and reduced β-sheet conformation as revealed by CD analysis, both of which show that NQTrp and Cl-NQTrp efficiently disassembled pre-formed PHF6 peptide fibrils. Further, to calculate the IC_50_ value (disassembly of 50% fibrils) for each hybrid molecules during fibril disassembly, we performed ThS assay with PHF6 in presence NQTrp and Cl-NQTrp at various molar ratios (Fig. 4a). We found that the IC_50_ values of NQTrp and Cl-NQTrp for disassembling fibrils from 50 µM PHF6 were 7 ± 1 and 6 ± 1 molar excess, respectively.

### Hybrid molecules disassemble pre-formed PHF6 fibrils to non-toxic intermediates

Oligomers of the tau protein were shown to be more toxic than the matured amyloid fibrils in the amyloidogenic pathway, since they can easily damage the cell wall resulting in neurodegeneration^14,36^. To verify whether disassembly of PHF6 fibrils facilitated by the hybrid molecules produced toxic species or not, we performed dye leakage assay using carboxyfluorescein entrapped large unilamellar vesicles (LUVs)^37–39^, which mimics the cell membrane. Prior to the leakage assay, the formation and integrity of the LUVs were confirmed by TEM analysis (Fig. 4b). To perform the vesicle leakage assay, we prepared samples containing untreated LUVs i.e., without PHF6 as negative control and LUVs incubated with PHF6 oligomer as positive control, in addition to test samples containing various disassembled PHF6 fibril preparations. NQTrp and Cl-NQTrp were added to pre-formed PHF6 fibrils and allowed to incubate for fibril disassembly as mentioned earlier. Thereafter, different preparations were added separately to the LUVs, maintaining a lipid to PHF6 molar ratio as 1:20. The four hours aged PHF6 was assumed to be mature fibrils, whereas freshly prepared PHF6 (emerging from 15–20 minutes into aggregation) was considered as oligomers. Triton X-100 (non-ionic surfactant) was used for the complete dye release from the LUVs. The final fluorescence readout was measured according to equation (1)^5^.

1 $$ \% \,{\rm{Leakage}}=\frac{({\rm{observed}}\,{\rm{fluorescence}}-{\rm{initial}}\,{\rm{fluorescence}})}{({\rm{total}}\,{\rm{fluorescence}}-{\rm{initial}}\,{\rm{fluorescence}})}\times 100 \% $$

We noticed that the PHF6 oligomers; which were prepared fresh and mixed imediately with the LUVs caused rapid and steep increment of dye leakage until 150 min and this increment slowly saturated after 200 minutes (red, Fig. 4c–f). However, the natural leakage of dye from LUVs (background fluorescence) was minimal and saturated after 100 minutes (black, Fig. 4c–f). This result indicated that the PHF6 oligomers significantly ruptured the LUVs causing the dye leakage, which demonstrated it to be toxic. On the other hand, lesser dye leakage was observed from the PHF6 aggregates treated with NQTrp or Cl-NQTrp, indicating these disassembled species was comparatively less toxic than the oligomeric preparations of PHF6 (cyan, green, pink, Fig. 4c–f). Corroborating the results from several other *in vitro* experiments mentioned above, a dose-dependence phenomenon was also observed in the dye leakage assay, elucidating a molar ratio of 1:5 [PHF6:NQTrp/Cl-NQTrp] as effective in fibril disassembly. Therefore, with this outcome, we concluded that the hybrid molecules i.e., NQTrp and Cl-NQTrp disassembled the pre-formed PHF6 fibrils to non-toxic intermediates.

### Hybrid molecules induce conformational changes in PHF6 oligomer

**Figure 5:**
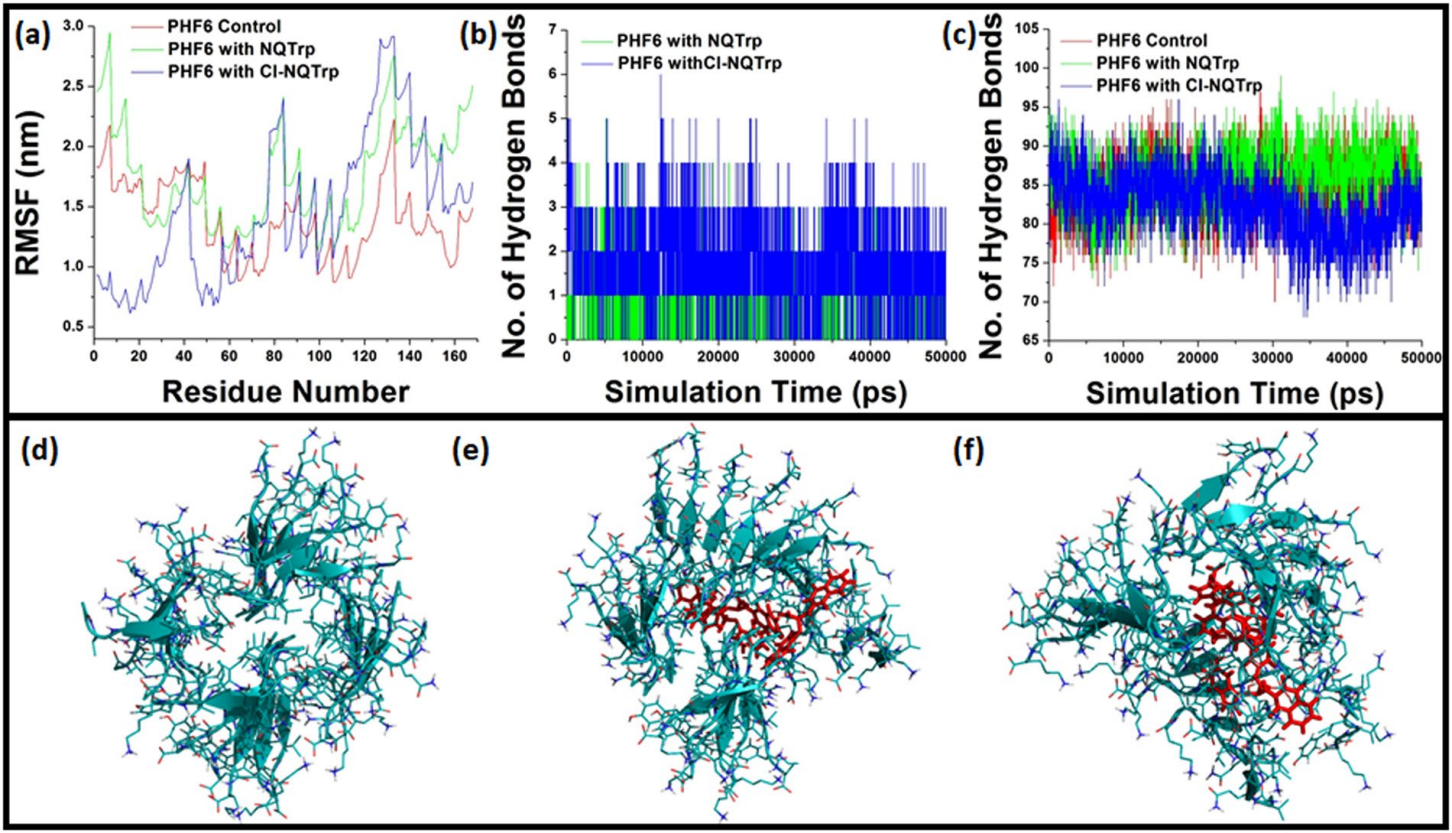
Geometric parameter analysis of oligomer system: (**a**) Comparative Root Mean Square Fluctuation (RMSF) analysis as a function of amino acid residues in the PHF6 oligomer. (**b**) Evolution of hydrogen bonds between NQTrp or Cl-NQTrp molecules with PHF6 peptides. (**c**) Reduction in main chain hydrogen bonds between PHF6 peptides in the presence of NQTrp or Cl-NQTrp. MD trajectories obtained after 50 ns simulation of the PHF6 oligomer with and without NQTrp or Cl-NQTrp. (**d**) Twisted control oligomer containing intact β-sheets in the absence of hybrid molecules, and in the presence of (**e**) NQTrp or (**f**) Cl-NQTrp causes loosely packed β-sheet structures and oligomer disassociation.

To obtain an atomistic view into the interaction of Naphthoquinone-Tryptophan hybrids with the oligomer and the fibril of PHF6 peptide during disassembly, we performed molecular dynamics (MD) simulations. Since heparin-induced aggregation of PHF6 is rapid *in vitro* (20 min), it is difficult to test whether the hybrid molecules can interfere with the process of oligomerization. Therefore, the attempt was made *in silico*, where we have more control over the process. The oligomer system was built using 24 PHF6 peptides in pre-defined β-sheet conformation, arranged in four strands with six peptides per strand^40^. To examine the structural stability of oligomer in the absence and the presence of the hybrid molecules, we calculated the C_α_-root mean square fluctuation (RMSF) as a function of the residue number over 50 ns MD simulation using *g_rmsf*. As shown in Fig. 5a, the RMSF value was constantly changing when the hybrid molecules interacted with the oligomer, which suggested that NQTrp and Cl-NQTrp likely have a prominent role in the conformational changes. Out of the four strands, residues 126–168 of the last strand underwent significant fluctuations when compared to the oligomers in the absence of the hybrid molecules. Also, the presence of NQTrp and Cl-NQTrp caused minor changes in RMSF of residues 75–90 of the second and third strand, respectively. Both hybrid molecules have hydrophobic moiety and side chains favoring hydrogen bond formation. The earlier study suggests that inhibitory molecules such as flavonoids, possessing hydrogen bonding capacity could be a significant factor for fibril destabilization^41^. In addition, interaction of protein with ligands such as polyphenols are strongly driven by hydrophobic effects which are stabilized by hydrogen bonding^42–44^. To identify the mechanism behind NQTrp and Cl-NQTrp interaction with the PHF6 oligomer, we calculated the evolution of hydrogen bonds during simulation using *g_hbond* and number of interaction using *g_mindist*. As the number of interaction increased, (Supplementary Fig. 2) single molecule of NQTrp or Cl-NQTrp formed an average of 1–2 hydrogen bonds with the peptide oligomer (Fig. 5b).

Experimental and theoretical evidence supports the notion that PHF6 peptide fibrils are made up of highly ordered cross β structures^26,45,46^. Moreover, we had previously examined the integrity, stability, and reduction in the β-sheet content of PHF6 assemblies following its inhibition with NQTrp or Cl-NQTrp^31,32^. We hypothesized that formation of hydrogen bonds between hybrid molecules and the peptide oligomers might reduce the interaction between adjacent peptides and neighboring oligomer strands. Since inter-peptide main chain hydrogen bonds govern the architecture of β-sheet conformation, it becomes essential to estimate the number of main chain hydrogen bonds during MD simulation with the hybrid molecules. To validate this, we calculated the number of main chain hydrogen bonds between the peptides in PHF6 control oligomers and found it to be stable throughout 50 ns simulation. However, in the presence of the hybrid molecules, there was a reduction in the main chain hydrogen bonds suggesting that interaction of NQTrp and Cl-NQTrp with the peptides may have disrupted inter-peptide hydrogen bonds responsible for holding the oligomers together (Fig. 5c). To further evaluate the structure of oligomer in the presence and absence of the hybrid molecules, we visualized the 3D architecture of the peptides using *trjconv*. Initial conformations of the oligomers at 0 ns are provided in the supplementary information (Supplementary Fig. 3). As shown in Fig. 5d, in the absence of the hybrid molecules the PHF6 oligomer had limited twisting and intact β-sheets without disassembly. On the other hand, the presence of NQTrp and Cl-NQTrp lead to loosely packed β-sheets and strands parted away when viewed from the central axis (Fig. 5e,f). This result corroborates the RMSF value which was attained for the last two strands of the oligomer simulated with the hybrid molecules (Fig. 5a). We also calculated the compactness of the oligomer system using *g_gyrate*. In the absence of the hybrid molecules, the radius of gyration (RoG) remained constant in the first 20 ns of the simulation suggesting that the oligomer was compact and stable (Supplementary Fig. 4). In contrast, in the presence of NQTrp or Cl-NQTrp, RoG values fluctuated and remained higher when compared to the control indicating that the peptides were moving away from each other, which was also noticeable from their trajectories. Taken together these results strongly indicate that NQTrp and Cl-NQTrp have the potential to induce conformational changes in PHF6 peptide oligomers.

### Complexation of NQTrp and Cl-NQTrp with PHF6 mediates the fibril disassembly

**Figure 6:**
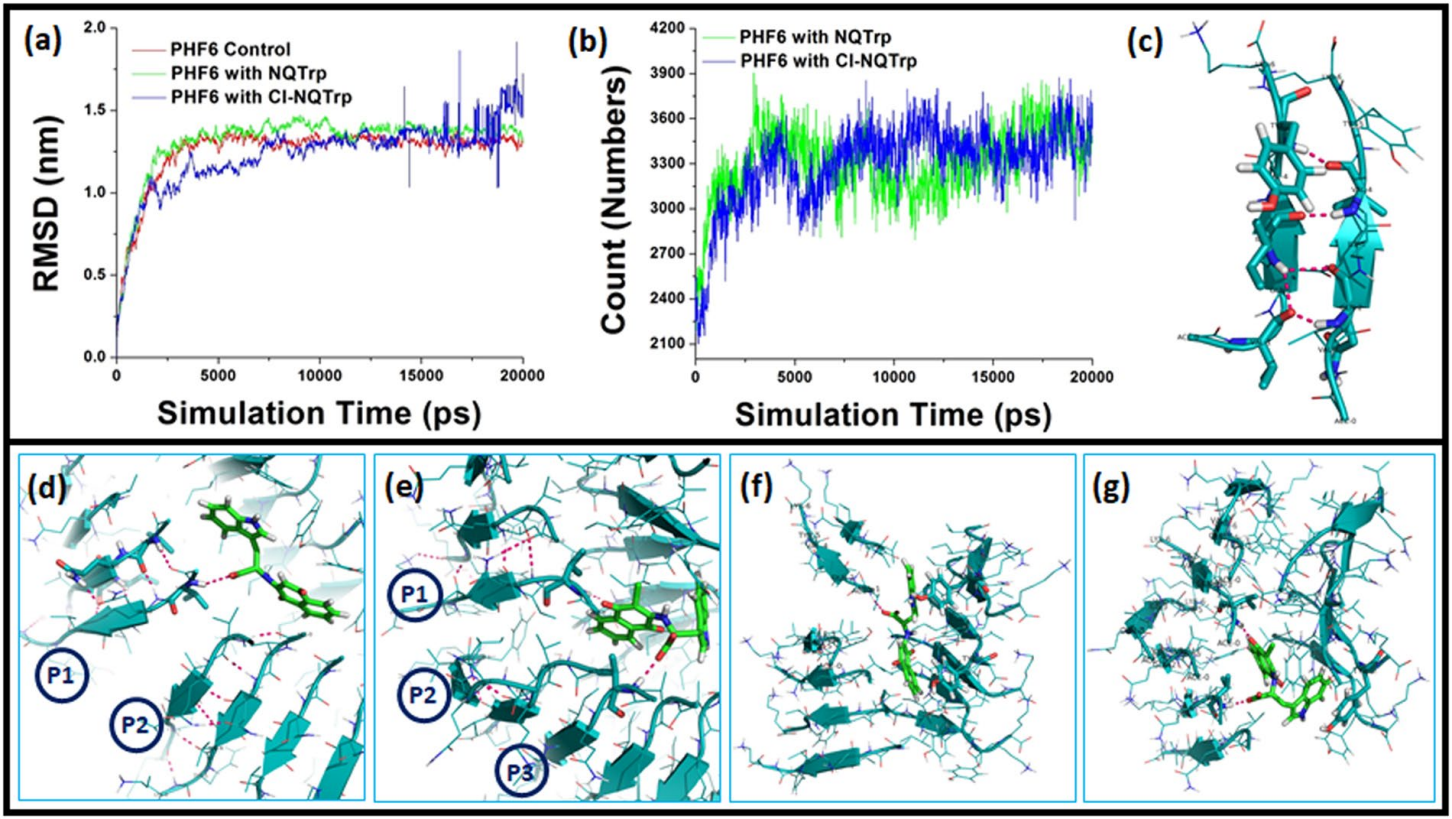
Geometric parameter analysis of PHF6 fibril system: (**a**) Plot of Root Mean Square Deviation (RMSD) vs. simulation time, (**b**) Number of interactions between NQTrp or Cl-NQTrp molecules and the peptides of PHF6 fibril calculated as a function of simulation time, (**c**) PHF6 peptide dimers in β-sheets conformation resulting from hydrogen bonds between amino acid residues, (**d**) NQTrp forms one hydrogen bond with Val_1_ of P1-PHF6 fibril disrupting the β-sheet between peptide pairs P1-P2, (**e**) Cl-NQTrp forms two hydrogen bonds: one with Val_1_ of P1 and another with Val_1_ of P3, causing disruption of the β-sheet between peptide pairs P1-P2. π-π stacking between (**f**) NQTrp and (**g**) Cl-NQTrp and the Tyr residue of PHF6 peptide fibril. Trajectories were recorded after 20 ns simulation. All interacting residues are shown as sticks. Green sticks represent hybrid molecules. Hydrogen bonds are shown as pink dashes. P1, P2 and P3 denote peptides of interest.

The PHF6 fibril system was built using 42 peptides in pre-defined β-sheet conformation, arranged in 2 strands with 21 peptides per strand. This design was optimized such that the fibril could give a complete twist during MD simulation. The conformational stability of the fibril system was monitored by the progression of C_α_-root mean square deviation (RMSD) as a function of time over 20 ns MD simulation using *g_rms*. The overall structure of the PHF6 fibril in the presence of NQTrp or Cl-NQTrp was changing as evident by the differences in RMSD value (Fig. 6a). During the initial stages of simulation (5 ns), when the fibril was simulated in the presence of Cl-NQTrp a lower deviation (~0.18 nm) was observed from the control, i.e., in the absence of the hybrid molecules. However, this deviation strikingly rose higher after 15 ns depicting a substantial conformational change in the system. RMSD of the PHF6 fibril in the presence of NQTrp was reasonably constant throughout the simulation and always stayed higher than the control fibril.

To compute the number of interactions between the hybrid molecules and the PHF6 fibril, we calculated the count as a function of simulation time. As seen in Fig. 6b, the hybrid molecules were at constant interaction with the fibril, and the number of interaction increased gradually from 0 ns to 20 ns. Since the interaction increased in due course of the simulation we asked by what mechanism the hybrid molecules found contact with the peptides in the fibril system. To understand this, we first simulated two PHF6 peptides in the absence of any hybrid molecule and observed that the β-sheets between these peptides were formed owing to the hydrogen bonds between main-chain of Val^1^_1_-Gln^2^_2_, Ile^1^_3_-Gln^2^_2_, Ile^1^_3_-Val^2^_4_ and Tyr^1^_5_-Val^2^_4_ (Fig. 6c). As in case of oligomer system, we hypothesized that interaction of the hybrid molecules with these hydrogen bond forming residues might disrupt the peptide-peptide interaction, eventually breaking the β-sheets. From the fibril simulation, we inferred that NQTrp and Cl-NQTrp predominantly interacted with hydrophobic amino acid Val, which was one of the key residues in maintaining the β-sheet conformation between two peptide pairs. A close look at the MD trajectory revealed that NQTrp formed one hydrogen bond with the main chain of Val_1_ of P1 (bond length 2.6 Å) (Fig. 6d). As seen in the figure, the adjacent peptides had undisrupted hydrogen bonds, but the interaction of NQTrp with P1 broke the inter main-chain hydrogen bonds between Val^P1^_1_-Gln^P2^_2_, Ile^P1^_3_-Gln^P2^_2_, Ile^P1^_3_-Val^P2^_4_ and Tyr^P1^_5_-Val^P2^_4_ of P1 and P2. On the other hand, Cl-NQTrp formed two main chain hydrogen bonds, one with Val_1_ of P1 (bond length 1.7 Å) and another with Val_1_ of P3 (bond length 2.1 Å) of the peptides in the fibril (Fig. 6e). Cl-NQTrp formed two hydrogen bonds owing to the fact that addition of Cl to the quinone ring increased the π-electron density and stabilized the enolate ion of the quinone ring, through election delocalization. This phenomenon provides an extra hydrogen bonding site in Cl-NQTrp which is absent in NQTrp. As in the case of NQTrp, the formation of hydrogen bonds between PHF6 peptides and Cl-NQTrp disrupted the inter main-chain hydrogen bonds between Val^P1^_1_-Gln^P2^_2_, Ile^P1^_3_-Gln^P2^_2_, Ile^P1^_3_-Val^P2^_4_ of P1 and P2. These results validate our hypothesis and demonstrate that ligand interaction with key residues of PHF6 responsible for β-sheet formation will disrupt the inter-peptide hydrogen bonds. Previously, it was reported that quinone-based molecules tend to intercalate between the hydrophobic residues and inhibit the process of β-sheet formation^31,47^. In addition, *Berthoumieu et al*. discussed the interaction between aromatic ring of Phe residues and NQTrp, which modulated the kinetics of aggregation^48^. To examine whether during fibril disassembly hybrid molecules have the ability to intercalate, we analyzed the trajectories to visualize the stacking of the molecules. As shown in Fig. 6f (and Supplementary Fig. 5) the aromatic ring of naphthoquinone and Trp of NQTrp formed π-π stacking with side chain of two Tyr residues of PHF6 peptide fibrils likewise, the aromatic ring of Trp of Cl-NQTrp formed π-π stacking with side chain of one Tyr residue (Fig. 6g, Supplementary Fig. 6). These hydrophobic interactions may further aid in disassembly by causing steric hindrance and untwisting of fibrils.

**Figure 7:**
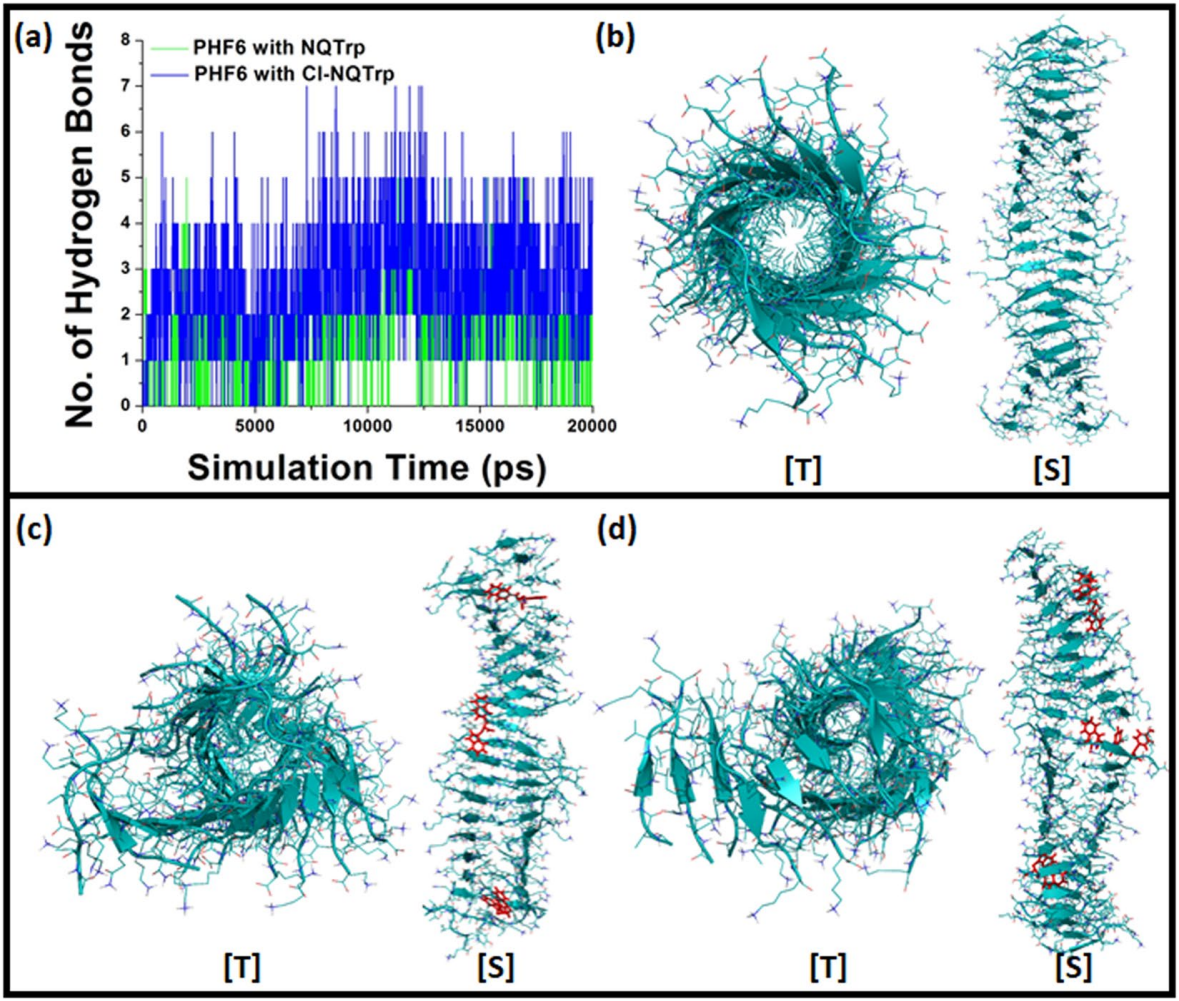
(**a**) Evolution of hydrogen bonds between NQTrp or Cl-NQTrp molecules with PHF6 peptide fibril. MD trajectories obtained after 20 ns simulation of the PHF6 fibril in the presence or in the absence (control) of the hybrid molecules: (**b**) Twisted control fibril containing β-sheets without disassembly; (**c**) NQTrp and (**d**) Cl-NQTrp caused loosely packed β-sheet structures and breaks in fibril strands. [T] denotes top view from the central axis and [S] denotes side view.

To establish a correlation between hydrophobic effects and stabilized interactions, we analyzed the evolution of hydrogen bonds between the hybrid molecules and the peptides in PHF6 fibril. Interestingly, we found that a single molecule of NQTrp or Cl-NQTrp could form an average of one to two hydrogen bonds with PHF6 fibril throughout the simulation (Fig. 7a). Our *in vitro* data showed that co-incubation of PHF6 fibrils with the hybrid molecules caused the reduction in the percentage of amyloid content by breaking pre-formed fibrils. To visualize whether NQTrp and Cl-NQTrp could cause physical ruptures in fibrillar strands, we analyzed the trajectories of the MD simulation (Fig. 7b–d). As seen in Fig. 7b, in the absence of the hybrid molecules (control) the fibril was intact without any evidence of disassembly. The top view from the central axis of the fibril revealed that the β-sheets were tightly packed, bonded well to each other, and the side view showed the uniform twist in fibril. On the other hand, when simulated in the presence of the hybrid molecules the conformation of the fibril drastically changed. As shown in Fig. 7c,d, fibril simulated with NQTrp or Cl-NQTrp had an unusual arrangement when viewed from central axis comprising of loosely packed β-sheets, which were parting away from each other. Clear breaks in the fibrillar strand were also noticed in places where the hybrid molecules contacted the peptides and formed hydrogen bonds with key residues. Both NQTrp and Cl-NQTrp caused breakage of PHF6 fibril at two regions; one on each fibrillar strand, leading to fibril disassembly. This data from MD simulation supports our *in vitro* assays by providing a proof-of-concept for a molecular mechanism of fibril disassembly by Naphthoquinone-Tryptophan hybrids.

### NQTrp and Cl-NQTrp effectively remodeled pre-formed aggregates of full-length tau protein

Recently reported cryo-electron microscopy structure of PHFs and SF revealed that the core of the tau filaments was composed of eight β-sheets (β1-8), i.e., β1-^306^VQIVYK^311^ (PHF6), β2-^313^VDLSKVTSKC^322^, β3-^327^NIHHK^331^, β4-^336^QVEVKS^341^, β5-^343^KLDFK^347^, β6-^349^RVQSKI^354^, β7-^356^SLDNITHV^363^, β8-^368^NKKIETHKLTF^378^ ^28^. These β-sheet structures run along the length of the proto-filament adopting a C-shaped architecture, where the N-terminal end of the ordered core is formed by the hexapeptide ^306^VQIVYK^311^ (β-1). The strategic location of β-1 is of prime importance as it forms a complementary packing interface with residues ^373^THKLTF^378^ of the β-8 and gives closure to the entire filament core of PHFs. As mentioned earlier, the hybrid molecules, i.e., NQTrp and Cl-NQTrp have strong affinity binding sites with PHF6 by forming hydrogen bonds with Val and hydrophobic interaction with Tyr, both of which together are essential hydrophobic residues of β-1 responsible in the formation of face-to-face packing with the hydrophobic groups of β-8 (Supplementary Fig. 7). To extend our hypothesis on fibril disassembly from the model system to full-length tau protein, we performed *in vitro* disassembly assays with wild type tau in the presence of differnt doses of NQTrp and Cl-NQTrp.

**Figure 8:**
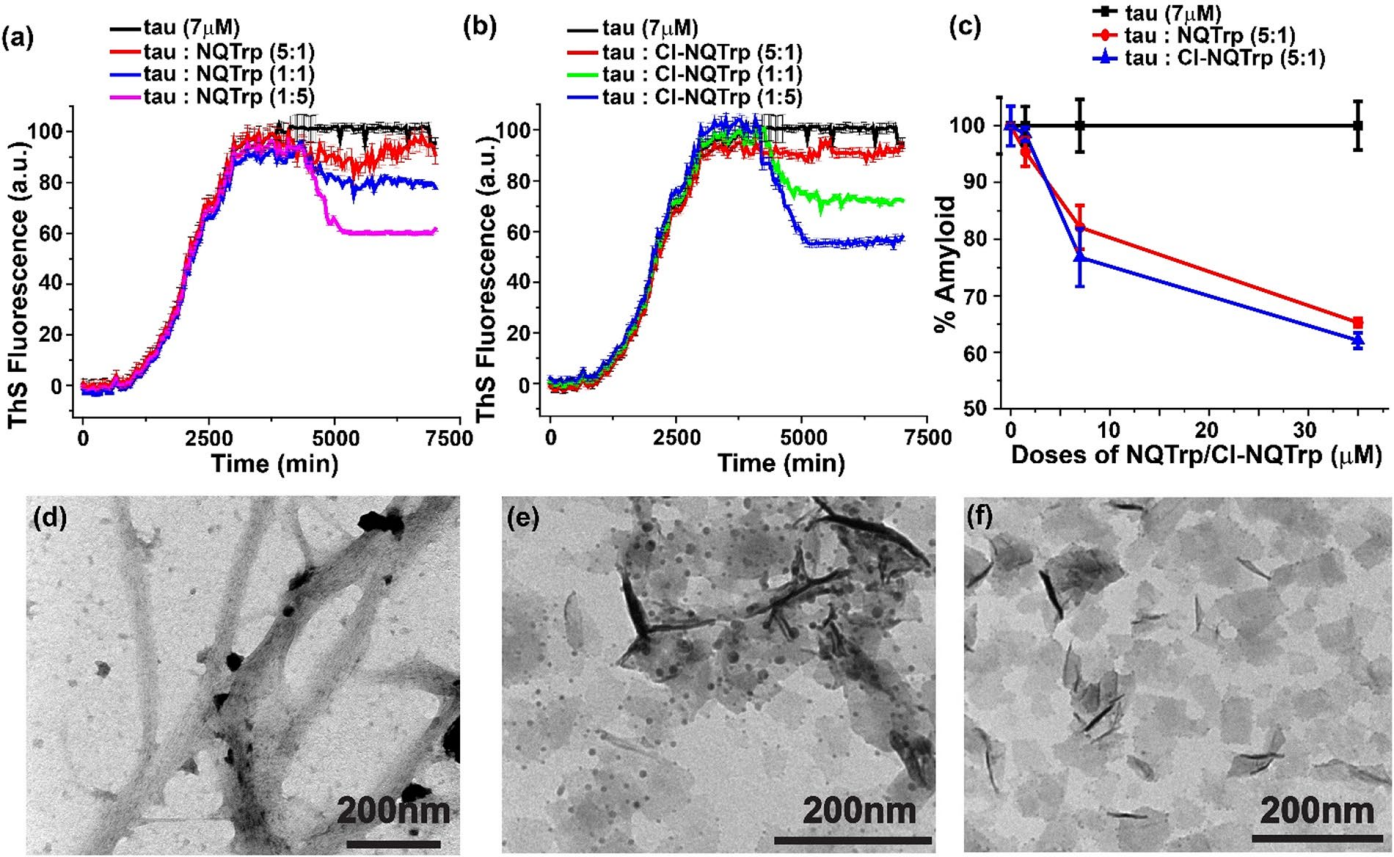
ThS fluorescence assay showing disassembly of pre-formed wild type tau protein aggregates in presence of (**a**) NQTrp (**b**) Cl-NQTrp. (**c**) Plot showing percentage amyloid remaining in the mixture after the fibril disassembly by NQTrp (red) and Cl-NQTrp (blue). TEM images showing disassembled wild type tau fibrils (**d**) in the absence and presence of 5 equivalent of (**e**) NQTrp and (**f**) Cl-NQTrp.

We first allowed the tau protein to aggregate in the absence of hybrid molecules (control) for 4000 minutes (black, Fig. 8a–c), where the real-time aggregation was moniotred by the increase in ThS fluorescence. Once a plateau in ThS fluorescence was achieved, differnt doses of NQTrp and Cl-NQTrp were added separately to the aggregated assembly and kinetics of disassembly process was monitored by the fall in ThS fluorescence for an additional 3000 minutes. Interestingly, we noticed that the results from disassembly of pre-formed tau aggregates by NQTrp and Cl-NQTrp mirror the outcome of PHF6 disassembly detailed earlier in Figs 2 and 3. This also validates the robustness of the assay and the cogency of our model system. As shown in Fig. 8a,b, a dose-dependent disassembly of pre-formed tau aggregates was visualized, where the drop in ThS fluorescence became significant with increased doses of the hybrid molecules. Maximum disassembly of fibrils was observed in presence of 5-fold molar excess NQTrp and Cl-NQTrp. To quantify the disassembly of tau fibrils by the hybrid molecules, a plot of percentage amyloids vs. doses of hybrid molecules was generated. Figure 8c indicates that ~35% of pre-formed fibrils were disrupted by 5-fold molar excess of NQTrp, whereas same molar quantity of Cl-NQTrp disrupted ~38%. Results from the ThS assay were further supported by TEM analysis. We visualized a clear fibrillar assembly of control tau protein i.e., in the absence of hybrid molecules (Fig. 8d). However, when a treatment with 5-fold molar excess of hybrid molecules was given to the pre-formed aggregates, no such fibrillary morphology was observed (Fig. 8e,f). Given that both NQTrp and Cl-NQTrp have high binding affinities with PHF6, the interaction between the hybrid molecules and pre-formed tau aggregates could plausibly open up the β-sheet core and this might be the reason why these molecules could efficiently disassemble pre-formed tau aggregates.

## Conclusion

Alzheimer’s disease (AD) is characterized by amyloid deposits of intra-cellular tau protein, i.e., NFTs and extra-cellular Aβ peptide, i.e., amyloid plaques. As a treatment strategy it becomes vital to develop compounds of dual attributes (i) inhibiting amyloid formation, which will stop the disease progression, and (ii) lessening pre-formed aggregates thus reducing the amyloid load. Along these lines, we have attempted to demonstrate the ability of Naphthoquinone-Tryptophan hybrids to disassemble pre-formed fibrils using PHF6 peptide as a model. Employing experimental and computational approaches, we show for the first time that NQTrp and Cl-NQTrp can disassemble pre-formed PHF6 peptide fibrils and full-length tau protein aggregates in a dose-dependent manner. As a plausible mechanism of disassembly, we deduced that the molecules’ interaction with β-sheet-rich fibrillar strand was mediated through hydrophobic effects in combination with the formation of hydrogen bonds with the key residues responsible for β-sheet formation between the peptide pairs. Interestingly, simulation results validated the *in vitro* data demonstrating the importance of *in silico* approach in providing mechanistic insights into disassembly of amyloid fibrils. Our findings underscore the dual nature of Naphthoquinone-Tryptophan hybrids as a scaffold for designing novel AD therapeutics.

## Experimental Methods

### Materials

All chemicals and reagents were of analytical grade. Unless otherwise stated, all chemicals were obtained from Sigma-Aldrich (Rehovot, Israel). Synthetic PHF6 peptide (Ac-VQIVYK-NH_2_) was purchased from GL Biochem (Shanghai, China). Cl-NQTrp was synthesized as described previously^49^.

### Stock preparation

PHF6 peptide was prepared for the aggregation assays as detailed by Frenkel-Pinter *et al*.^31^. In brief, peptides were monomerized by a 10 min pre-treatment with HFIP and the solvent was evaporated using a SpeedVac. The resulting thin film was dissolved in ddH_2_O and sonicated for 5 min. Concentration of the peptide was determined (calculated according to ε_280_ of 1490 M^−1^cm^−1^) and adjusted to 1 mM concentration with 20 mM MOPS (pH 7.2) as a stock solution. Stock solutions of Thioflavin S (ThS, 4 mM,) and heparin (1 mM) were prepared in 20 mM MOPS (pH 7.2). Stocks of hybrid molecules (100 mM) were prepared by dissolving NQTrp and Cl-NQTrp in DMSO, separately.

### PHF6 fibril disassembly assay

For self-assembly experiments, stock solutions were diluted in 100 µL wells of a 96-well black plate so that the final mixture contained 50 µM of the peptide and 100 µM ThS in 20 mM MOPS. Immediately prior to the experiment, heparin (10 µM) was added to initiate peptide aggregation. For fibril disassembly assays, PHF6 was first allowed to self-assemble as mentioned above. When a plateau of ThS fluorescence was attained (~60 min), NQTrp or Cl-NQTrp (ratio PHF6: NQTrp/Cl-NQTrp; 5:1, 1:1, 1:2, 1:5) was added separately to designated wells, and the assay was further continued. Control wells were supplemented with the same concentration of DMSO as test wells that contained the compounds. Kinetic fluorescence data were collected at 25 °C in quadruplicate using Infinite M200 microplate reader (Tecan, Switzerland), with measurements taken at 20 s intervals for 60 min (self-assembly) and 40 min (disassembly). Excitation and emission wavelengths of ThS were 440 and 490 nm, respectively.

### Disassembly of full-length tau protein aggregates

Wild type tau (441 amino acid residues, mw-45 kDa) was purchased from Anaspec, USA and used directly without further purification. Tau protein was dissolved in 50 mM PBS, pH 7.4 (137 mM NaCl, 3 mM KCl, 10 mM Na_2_HPO_4_, 2 mM KH_2_PO_4_) yielding a final tau concentration of 7 µM. Aggregation assay was performed at 37 °C in the presence of an anionic cofactor heparin (MW ≈ 6000 Da), used in a molar ratio of tau/heparin of 4:1^50^. The aggregation kinetics was monitored by ThS fluorescence in 384 black well-plate with 25 µL well volume. Kinetic fluorescence data were collected in triplicate using Infinite M200 microplate reader (Tecan, Switzerland), with measurements taken at 15 min intervals for 4000 min (assembly). When a plateau of ThS fluorescence was attained, NQTrp or Cl-NQTrp (ratio tau: NQTrp/Cl-NQTrp; 5:1, 1:1, 1:5) was added separately to designated wells, and the assay was further continued for 3000 min (disassembly). Excitation and emission wavelengths of ThS were 440 and 490 nm, respectively.

### Circular dichroism Spectroscopy

To analyze the secondary structure of disassembled fibrils, PHF6 peptide (50 µM) was allowed to self-assemble for 2 h at 25 °C in the absence of compound and further incubated for 3 h with 50 µM and 250 µM of NQTrp or Cl-NQTrp. Since DMSO absorbs at far UV range, stocks of hybrid molecules were prepared in methanol for this assay. Samples were placed in a 1 mm cuvette, and CD spectra were then recorded on a Chirascan spectrometer between the range of 200–250 nm, and the background was subtracted from the CD spectra.

### Transmission Electron Microscopy

Disassembled PHF6 peptide fibrils were prepared as mentioned in fibril disassembly assay. Samples (10 µL) were placed for 2 min on 400-mesh copper grids covered with carbon-stabilized Formvar film (Electron Microscopy Sciences (EMS), Hatfield, PA). Excess fluid was removed, and the grids were negatively stained with 2% uranyl acetate solution (10 µL) for 2 min. Finally, excess fluid was removed, and the samples were viewed using a JEM-1400 TEM (JEOL), operated at 80 kV.

### Large Unilamellar Vesicles (LUVs) preparation and Carboxyfluorescein entrapment

The vesicles were prepared as described earlier^37,38^. Briefly, the LUVs were prepared using three different lipids, DMPC, Cholesterol and GM1 with 68:30:2 molar ratios in 20 mM MOPS buffer of pH 7.2. All the lipids were taken in clean glass vessel and solubilized to make 1 mM stock solution in chloroform and methanol (2:1) and the solvents were evaporated under vacuum. The lipid films were hydrated with 620 µL of carboxyfluorescein solution (100 µM) in 20 mM MOPS buffer and immediately vortexed vigorously for 40 min to emulsify the lipid mixtures. Then, lipid solution was dipped into the liquid nitrogen for instant cooling and after 2 min the frozen solution was dipped into water bath at 50–60 °C for thawing^37^. These step of freeze-thaw was repeated five times and excess dye was removed by ultracentrifugation at 20000 rpm. The supernatant solution was discarded and the lipid pellet was re-hydrated with 20 mM MOPS. This step was repeated two more times and the final lipid pellet was collected followed by addition of 620 µL of MOPS buffer and vortexed to obtain homogenous suspension of 1 mM of dye loaded LUVs. The dye leakage study was performed in triplicate on Infinite M200 microplate reader (Tecan, Switzerland).

### Molecular Dynamics

The coordinates of PHF6 peptide were obtained from PDB ID: 2ON9^46^ and the N-terminus of the peptide was acetylated. The X-ray unit cell of 2ON9 was replicated to attain the oligomer structure consisting of 24 peptide units and fibril structure consisting of 42 peptide units. Three-dimensional conformer of NQTrp was obtained from PubChem (CID: 56605052), and Cl-NQTrp was generated by attaching a chloride group to NQTrp. GROMACS topology for the molecules were generated using the PRODRG server^51^. The details of the simulated systems are presented in (Supplementary Table 1). Molecular dynamics simulation was performed in the isothermal-isobaric ensemble using GROMACS (4.5.3) with GROMOS96 force field^52,53^. Water was described using the simple point charge (SPC) water model. The pressure was controlled at 1 atm and temperature was retained at 310 K using Parrinello-Rahman Barostat and V-rescale thermostat, respectively. A salt concentration of 0.15 M NaCl was added to mimic the physiological conditions. Two femtoseconds (fs) time step was used to integrate the equation of motion. The electrostatic interaction was calculated using Particle Mesh Ewald sums with a non-bonded cut off 10 Å. Bonds between hydrogen and heavy atoms were constrained at their equilibrium length using the linear constraint solver (LINCS) algorithm^54^. Initially, the energy minimization of the system was carried out followed by the equilibration for 100 picoseconds (ps). Subsequently, a production run for 50 ns and 20 ns was performed for oligomer and fibril system, respectively. Trajectories were saved at 10 ps intervals, and analyses of the trajectories were carried out using GROMACS suite of programs and PyMOL (http://www.pymol.org).

## Electronic supplementary material

Supplementary information

## References

[1] Walsh DM, Selkoe DJ (2004). Deciphering the molecular basis of memory failure in Alzheimer’s disease. Neuron.

[2] Bloom GS (2014). Amyloid-beta and tau: the trigger and bullet in Alzheimer disease pathogenesis. JAMA Neurol..

[3] Van Cauwenberghe C, Van Broeckhoven C, Sleegers K (2016). The genetic landscape of Alzheimer disease: clinical implications and perspectives. Genet. Med..

[4] Arriagada PV, Growdon JH, Hedley-Whyte ET, Hyman BT (1992). Neurofibrillary tangles but not senile plaques parallel duration and severity of Alzheimer’s disease. Neurology.

[5] Kosik KS (1993). The molecular and cellular biology of tau. Brain Pathol..

[6] Binder LI, Frankfurter A, Rebhun LI (1985). The distribution of tau in the mammalian central nervous system. J. Cell Biol..

[7] Kolarova M, García-Sierra F, Bartos A, Ricny J, Ripova D (2012). Structure and pathology of tau protein in Alzheimer disease. Int. J. Alzheimers. Dis..

[8] Frenkel-Pinter M (2016). Selective Inhibition of Aggregation and Toxicity of a Tau-Derived Peptide using Its Glycosylated Analogues. Chem. – A Eur. J..

[9] Yuzwa SA (2012). Increasing O-GlcNAc slows neurodegeneration and stabilizes tau against aggregation. Nat. Chem. Biol..

[10] Alonso A, Zaidi T, Novak M, Grundke-Iqbal I, Iqbal K (2001). Hyperphosphorylation induces self-assembly of tau into tangles of paired helical filaments/straight filaments. Proc. Natl. Acad. Sci. USA.

[11] Barghorn S, Davies P, Mandelkow E (2004). Tau paired helical filaments from Alzheimer’s disease brain and assembled *in vitro* are based on beta-structure in the core domain. Biochemistry.

[12] Gómez-Ramos A, Díaz-Hernández M, Cuadros R, Hernández F, Avila J (2006). Extracellular tau is toxic to neuronal cells. FEBS Lett..

[13] Guerrero-Muñoz MJ, Gerson J, Castillo-Carranza DL (2015). Tau Oligomers: The Toxic Player at Synapses in Alzheimer’s Disease. Front. Cell. Neurosci..

[14] Ward SM, Himmelstein DS, Lancia JK, Binder LI (2012). Tau oligomers and tau toxicity in neurodegenerative disease. Biochem. Soc. Trans..

[15] Santa-Maria I (2012). Paired helical filaments from Alzheimer’s disease brain induce intracellular accumulation of Tau in aggresomes. J. Biol. Chem..

[16] Spires-Jones TL, Hyman BT (2014). The intersection of amyloid beta and tau at synapses in Alzheimer’s disease. Neuron.

[17] Ng YP, Or TCT, Ip NY (2015). Plant alkaloids as drug leads for Alzheimer’s disease. Neurochem. Int..

[18] Chua SW (2017). The Polyphenol Altenusin Inhibits *in Vitro* Fibrillization of Tau and Reduces Induced Tau Pathology in Primary Neurons. ACS Chem. Neurosci..

[19] Wobst HJ, Sharma A, Diamond MI, Wanker EE, Bieschke J (2015). The green tea polyphenol (−)-epigallocatechin gallate prevents the aggregation of tau protein into toxic oligomers at substoichiometric ratios. FEBS Lett..

[20] Zhu M (2004). The flavonoid baicalein inhibits fibrillation of alpha-synuclein and disaggregates existing fibrils. J. Biol. Chem..

[21] Pickhardt M (2005). Anthraquinones inhibit tau aggregation and dissolve Alzheimer’s paired helical filaments *in vitro* and in cells. J. Biol. Chem..

[22] Paranjape SR (2015). Azaphilones inhibit tau aggregation and dissolve tau aggregates *in vitro*. ACS Chem. Neurosci..

[23] Caruana M (2011). Inhibition and disaggregation of alpha-synuclein oligomers by natural polyphenolic compounds. FEBS Lett..

[24] Du W-J (2015). Brazilin inhibits amyloid β-protein fibrillogenesis, remodels amyloid fibrils and reduces amyloid cytotoxicity. Sci. Rep..

[25] Ghosh A (2017). Inhibition and Degradation of Amyloid Beta (Abeta40) Fibrillation by Designed Small Peptide: A Combined Spectroscopy, Microscopy, and Cell Toxicity Study. ACS Chem. Neurosci..

[26] von Bergen M (2000). Assembly of tau protein into Alzheimer paired helical filaments depends on a local sequence motif ((306)VQIVYK(311)) forming beta structure. Proc. Natl. Acad. Sci. USA.

[27] von Bergen M (2001). Mutations of tau protein in frontotemporal dementia promote aggregation of paired helical filaments by enhancing local beta-structure. J. Biol. Chem..

[28] Fitzpatrick AWP (2017). Cryo-EM structures of tau filaments from Alzheimer’s disease. Nature.

[29] Mohamed T, Hoang T, Jelokhani-Niaraki M, Rao PPN (2013). Tau-Derived-Hexapeptide (306)VQIVYK(311) Aggregation Inhibitors: Nitrocatechol Moiety as A Pharmacophore In Drug Design. ACS Chem. Neurosci..

[30] Zheng J (2011). Macrocyclic beta-sheet peptides that inhibit the aggregation of a tau-protein-derived hexapeptide. J. Am. Chem. Soc..

[31] Frenkel-Pinter M (2016). Naphthoquinone-Tryptophan Hybrid Inhibits Aggregation of the Tau-Derived Peptide PHF6 and Reduces Neurotoxicity. J. Alzheimers. Dis..

[32] Frenkel-Pinter M (2017). Cl-NQTrp Alleviates Tauopathy Symptoms in a Model Organism through the Inhibition of Tau Aggregation-Engendered Toxicity. Neurodegener. Dis..

[33] Scherzer-Attali R, Shaltiel-Karyo R, Adalist YH, Segal D, Gazit E (2012). Generic inhibition of amyloidogenic proteins by two naphthoquinone-tryptophan hybrid molecules. Proteins.

[34] Iqbal K, Liu F, Gong C-X, Grundke-Iqbal I (2010). Tau in Alzheimer disease and related tauopathies. Curr. Alzheimer Res..

[35] Lasagna-Reeves CA (2011). Tau oligomers impair memory and induce synaptic and mitochondrial dysfunction in wild-type mice. Mol. Neurodegener..

[36] Flach K (2012). Tau oligomers impair artificial membrane integrity and cellular viability. J. Biol. Chem..

[37] Paul A, Kalita S, Kalita S, Sukumar P, Mandal B (2017). Disaggregation of Amylin Aggregate by Novel Conformationally Restricted Aminobenzoic Acid containing α/β and α/γ Hybrid Peptidomimetics. Sci. Rep..

[38] Williams TL, Day IJ, Serpell LC (2010). The effect of Alzheimer’s Abeta aggregation state on the permeation of biomimetic lipid vesicles. Langmuir.

[39] McLaurin J, Chakrabartty A (1996). Membrane disruption by Alzheimer beta-amyloid peptides mediated through specific binding to either phospholipids or gangliosides. Implications for neurotoxicity. J. Biol. Chem..

[40] Berhanu WM, Masunov AE (2015). Atomistic mechanism of polyphenol amyloid aggregation inhibitors: molecular dynamics study of Curcumin, Exifone, and Myricetin interaction with the segment of tau peptide oligomer. J. Biomol. Struct. Dyn..

[41] Lemkul JA, Bevan DR (2010). Destabilizing Alzheimer’s Abeta(42) protofibrils with morin: mechanistic insights from molecular dynamics simulations. Biochemistry.

[42] Gilson MK, Zhou H-X (2007). Calculation of protein-ligand binding affinities. Annu. Rev. Biophys. Biomol. Struct..

[43] Gohlke H, Kiel C, Case DA (2003). Insights into protein-protein binding by binding free energy calculation and free energy decomposition for the Ras-Raf and Ras-RalGDS complexes. J. Mol. Biol..

[44] Wiltzius JJW (2009). Molecular mechanisms for protein-encoded inheritance. Nat. Struct. Mol. Biol..

[45] Vaden TD (2008). Conformational preferences of an amyloidogenic peptide: IR spectroscopy of Ac-VQIVYK-NHMe. J. Am. Chem. Soc..

[46] Sawaya MR (2007). Atomic structures of amyloid cross-[beta] spines reveal varied steric zippers. Nature.

[47] Bulic B (2009). Development of tau aggregation inhibitors for Alzheimer’s disease. Angew. Chem. Int. Ed. Engl..

[48] Berthoumieu O (2015). Combined experimental and simulation studies suggest a revised mode of action of the anti-Alzheimer disease drug NQ-Trp. Chemistry.

[49] Shrestha-Dawadi PB, Bittner S, M F, S R (1996). On the synthesis of Naphthoquinonyl Hetrocyclic Amino Acids. Synthesis (Stuttg)..

[50] KrishnaKumar VG, Gupta S (2017). Simplified method to obtain enhanced expression of tau protein from *E. coli* and one-step purification by direct boiling. Prep. Biochem. Biotechnol..

[51] Schuttelkopf AW, van Aalten DMF (2004). PRODRG: a tool for high-throughput crystallography of protein-ligand complexes. Acta Crystallogr. D. Biol. Crystallogr..

[52] Lindahl E, Hess B, van der Spoel D (2001). GROMACS 3.0: a package for molecular simulation and trajectory analysis. Mol. Model. Annu..

[53] Schmid N (2011). Definition and testing of the GROMOS force-field versions 54A7 and 54B7. Eur. Biophys. J..

[54] Hess B, Bekker H, Berendsen HJC, Fraaije JGEM (1997). LINCS: A linear constraint solver for molecular simulations. J. Comput. Chem..

