# Supplementary material for: Mechanistic insights into remodeled Tau-derived PHF6 peptide fibrils by Naphthoquinone-Tryptophan hybrids
Source: Sci Rep. 2018 Jan 8;8:71. doi: 10.1038/s41598-017-18443-2 (PMC5758761; doi:10.1038/s41598-017-18443-2)

**Supplementary Information**

**Mechanistic insights into remodeled Tau-derived PHF6 peptide fibrils by Naphthoquinone-Tryptophan hybrids**

V. Guru KrishnaKumar,^#1, 2^ Ashim Paul,^#1^ Ehud Gazit,*^1,3^ and Daniel Segal*^1,4^

^[1]^ Department of Molecular Microbiology and Biotechnology, Tel Aviv University, Ramat Aviv, Tel Aviv 69978, (Israel)

^[2]^ Department of Biological Engineering, Indian Institute of Technology, Gandhinagar, Palaj, Gandhinagar, Gujarat 382355 (India)

^[3]^ Department of Materials Science and Engineering Iby and Aladar Fleischman Faculty of Engineering, Tel Aviv University, Ramat Aviv, Tel Aviv 69978, Israel

^[4]^ Interdisciplinary Sagol School of Neurosciences, Tel-Aviv University, Tel Aviv 69978 (Israel)

^#^ Authors contributed equally to this work

E-mail: [ehudg@post.tau.ac.il](mailto:ehudg@post.tau.ac.il), [dsegal@post.tau.ac.il](mailto:dsegal@post.tau.ac.il)

**Contents**

**Supplementary Figure 1**. The number of fibril present per square micrometer area was calculated from TEM images from each sample and their relative frequency vs. number of fibril was plotted

**Supplementary Figure 2**. Number of interactions between ligands and PHF6 oligomer determined as a function of simulation time.

**Supplementary Figure 3**. Initial conformation of PHF6 oligomers at 0 ns (a) PHF6 oligomer control (b) PHF6 oligomer with NQTrp (c) PHF6 oligomer with Cl-NQTrp

**Supplementary Figure 4**. Plot showing the Radius of gyration of PHF6 peptide oligomer system in the presence and absence of NQTrp and Cl-NQTrp

**Supplementary Figure 5.** π-π interaction between NQTrp and two Tyr residues of PHF6 peptide fibril. Green and orange stick represents NQTrp and side chain of Tyr, respectively.

**Supplementary Figure 6.** π-π interaction between Cl-NQTrp and Tyr residue of PHF6 peptide fibril. Green and pink stick represents Cl-NQTrp and side chain of Tyr, respectively.

**Supplementary Figure 7**. Representation of face-to-face packing of β-1 (^306^VQIVYK^311^) and part of β-8 (^373^THKLTF^378^) involved in the β-sheet structure of tau core filament.

**Supplementary Table 1.** Description of simulated systems


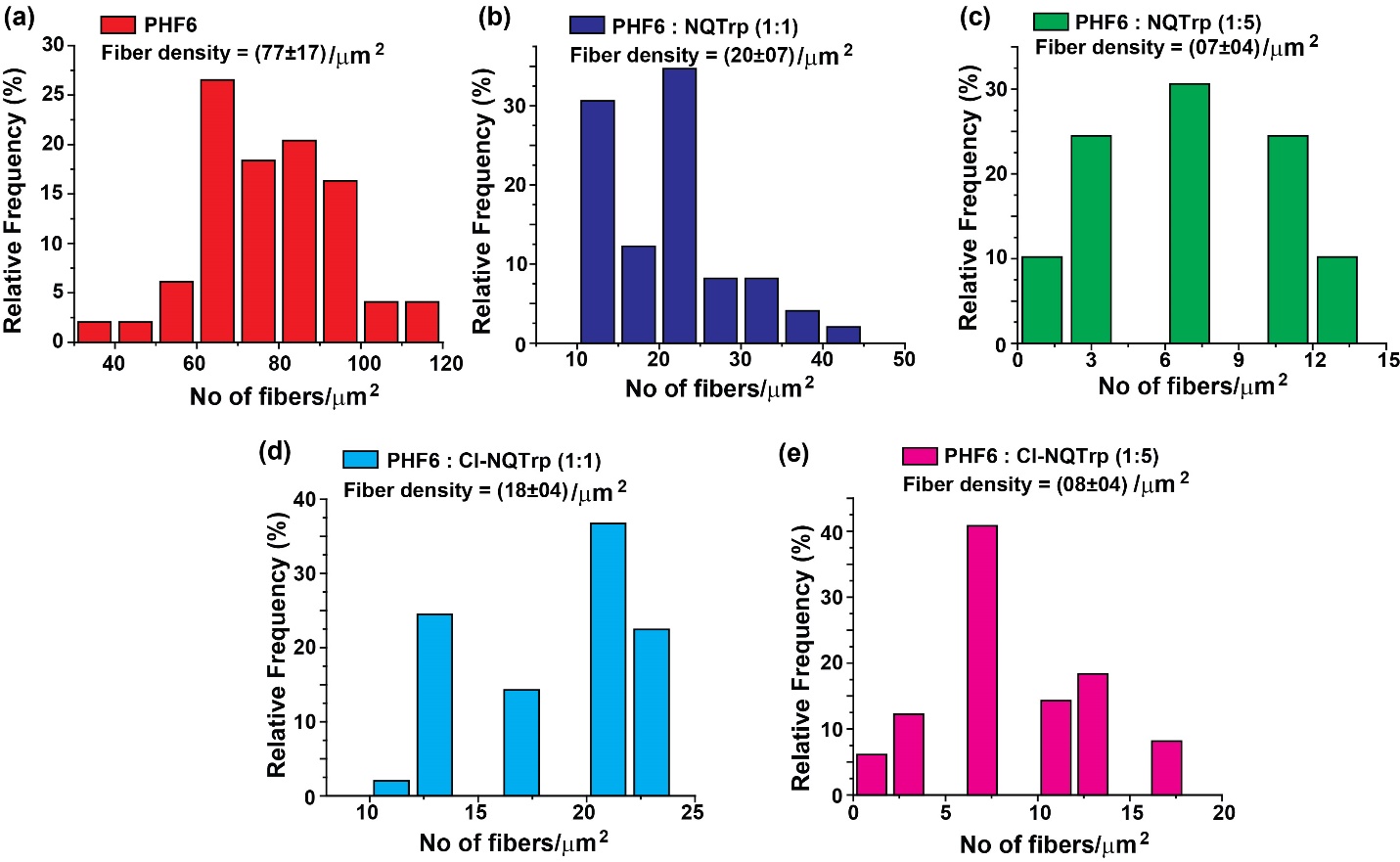


**Supplementary Figure 1**. The number of fibril present per square micrometer area was calculated from TEM images from each sample and their relative frequency vs. number of fibril was plotted. Average number of fibrils present per µm^2^ area of (a) 50 µM of PHF6 was (77±17), (b) PHF6:NQTrp (1:1) was (20±07), (c) PHF6:NQTrp (1:5) was (07±04), (d) PHF6:Cl-NQTrp (1:1) was (18±04) and PHF6:Cl-NQTrp (1:5) was (08±04).


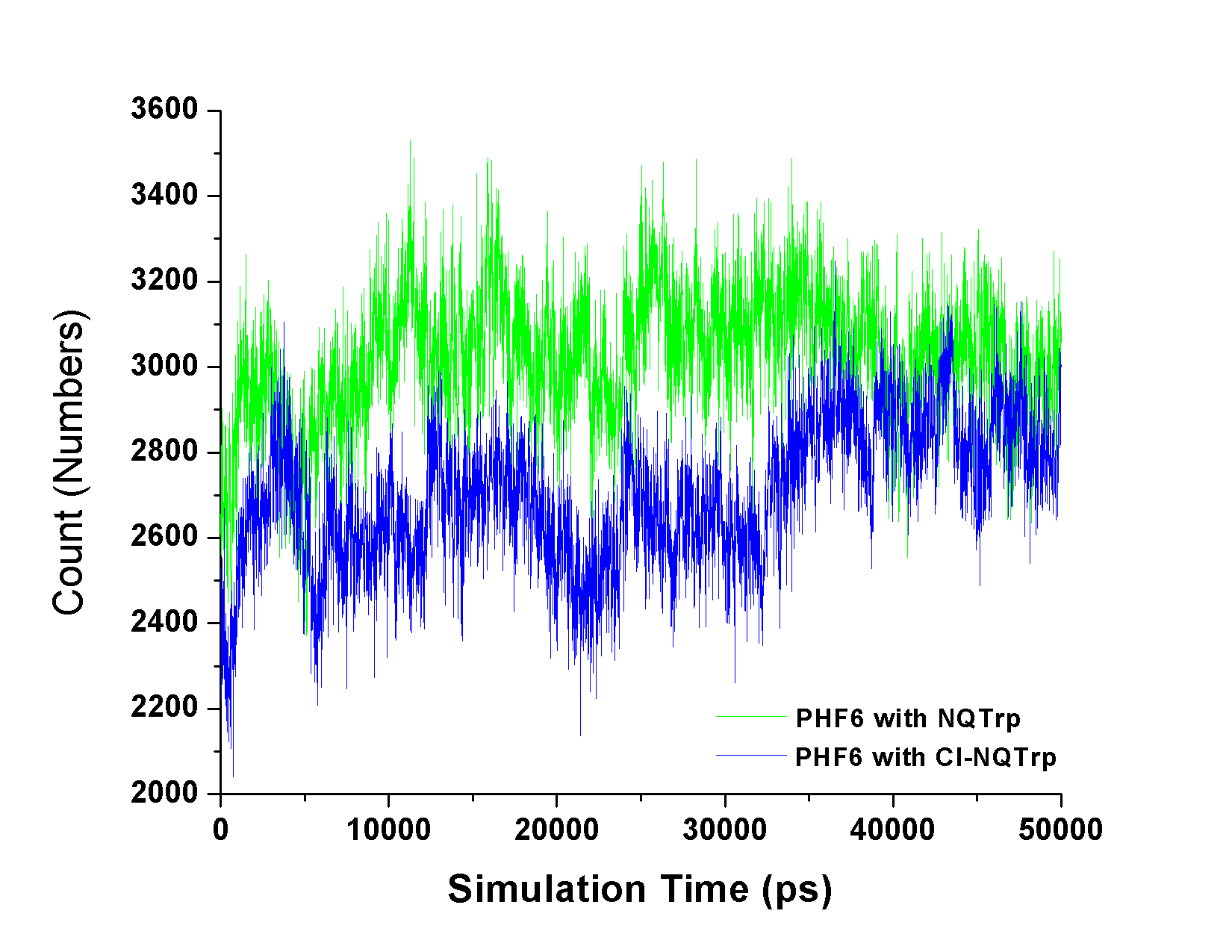


**Supplementary Figure 2**. Number of interactions between ligands and PHF6 oligomer determined as a function of simulation time.


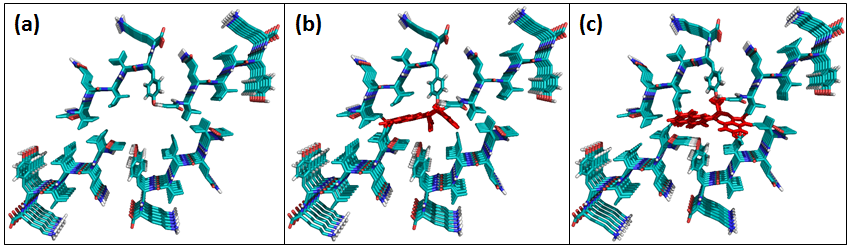


**Supplementary Figure 3**. Initial conformation of PHF6 oligomers at 0 ns (a) PHF6 oligomer control (b) PHF6 oligomer with NQTrp (c) PHF6 oligomer with Cl-NQTrp


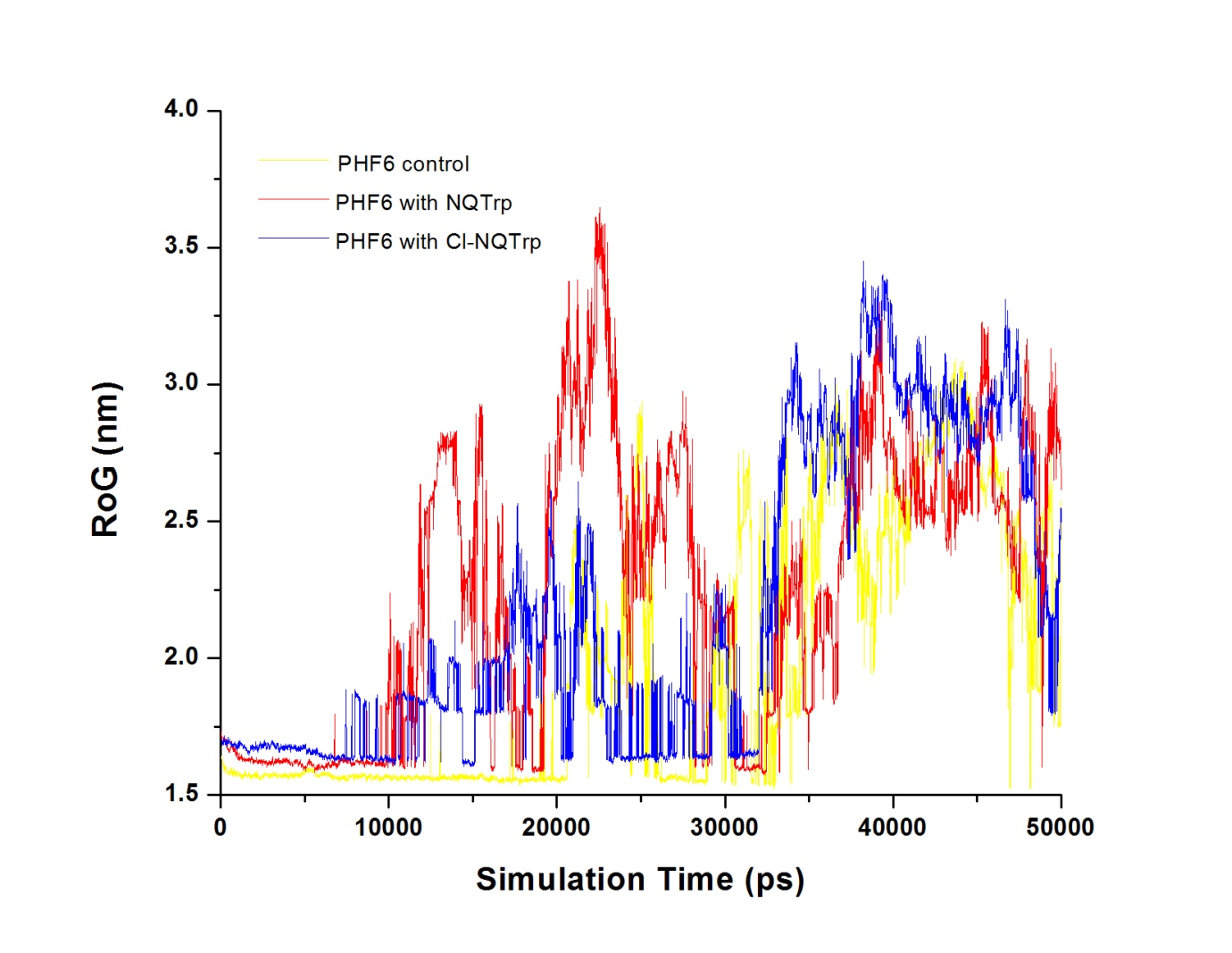


**Supplementary Figure 4**. Plot showing the Radius of gyration of PHF6 peptide oligomer system in the presence and absence of NQTrp and Cl-NQTrp


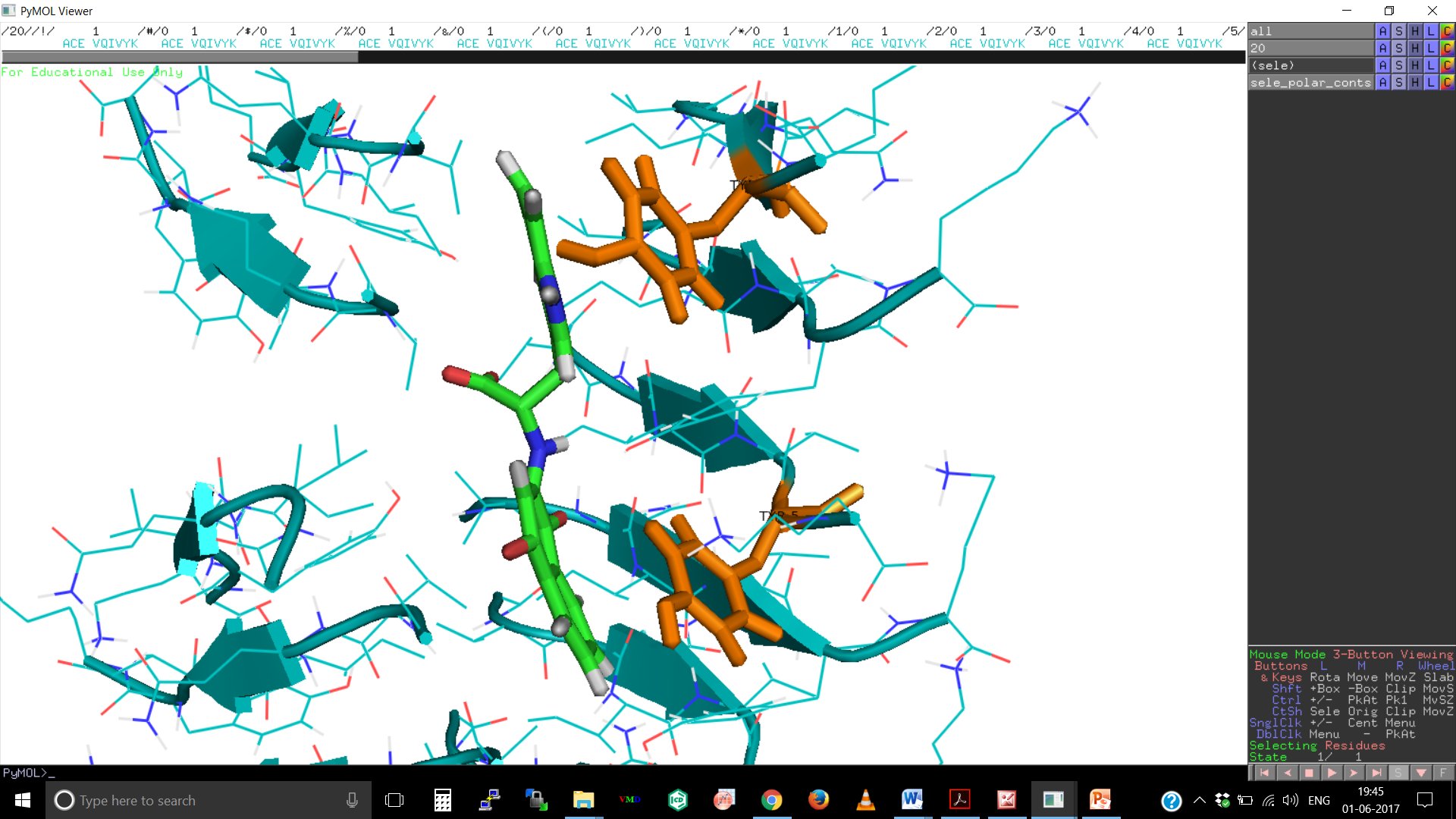


**Supplementary Figure 5.** π-π interaction between NQTrp and two Tyr residues of PHF6 peptide fibril. Green and orange stick represents NQTrp and side chain of Tyr, respectively.


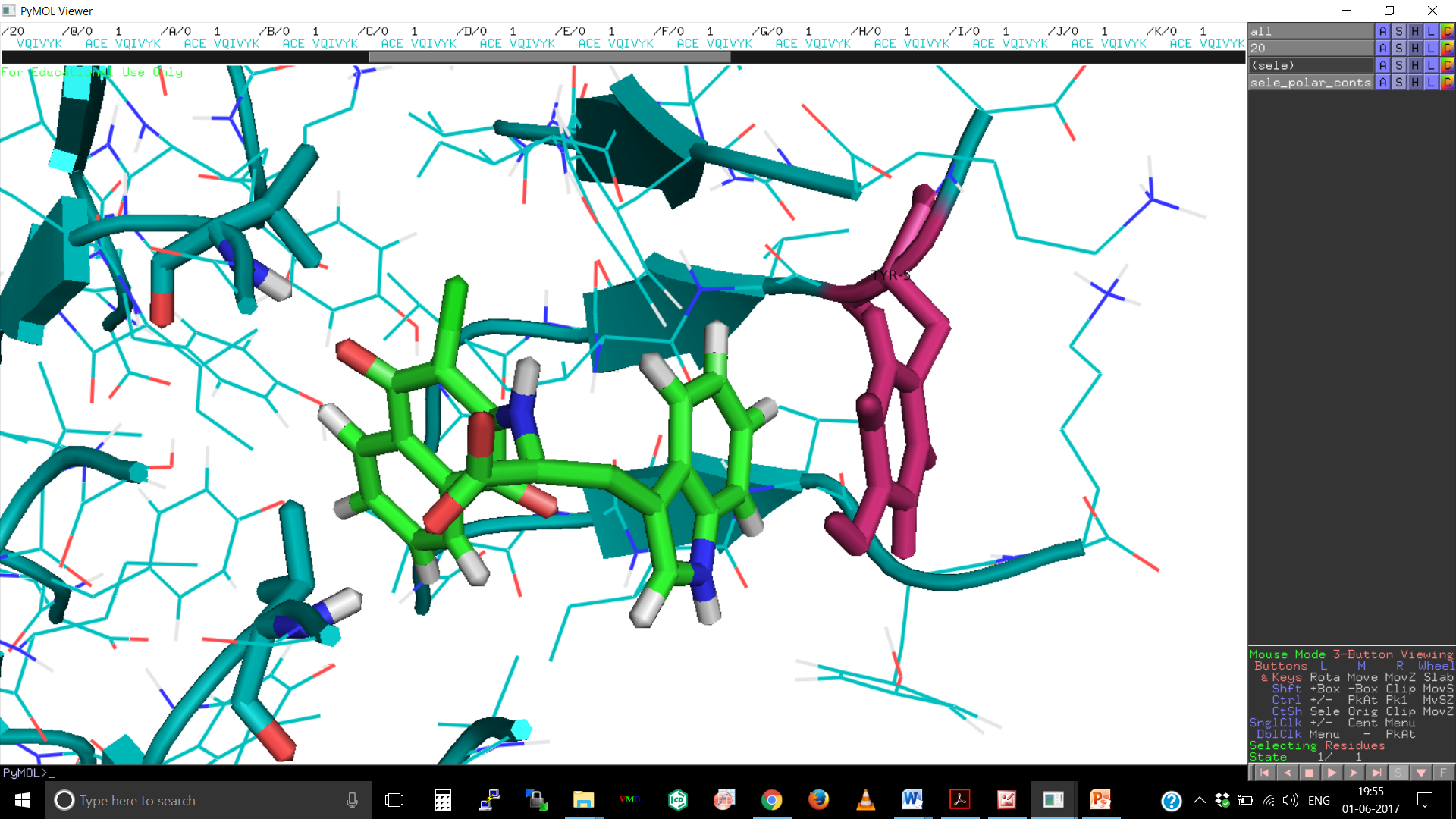


**Supplementary Figure 6.** π-π interaction between Cl-NQTrp and Tyr residue of PHF6 peptide fibril. Green and pink stick represents Cl-NQTrp and side chain of Tyr, respectively.


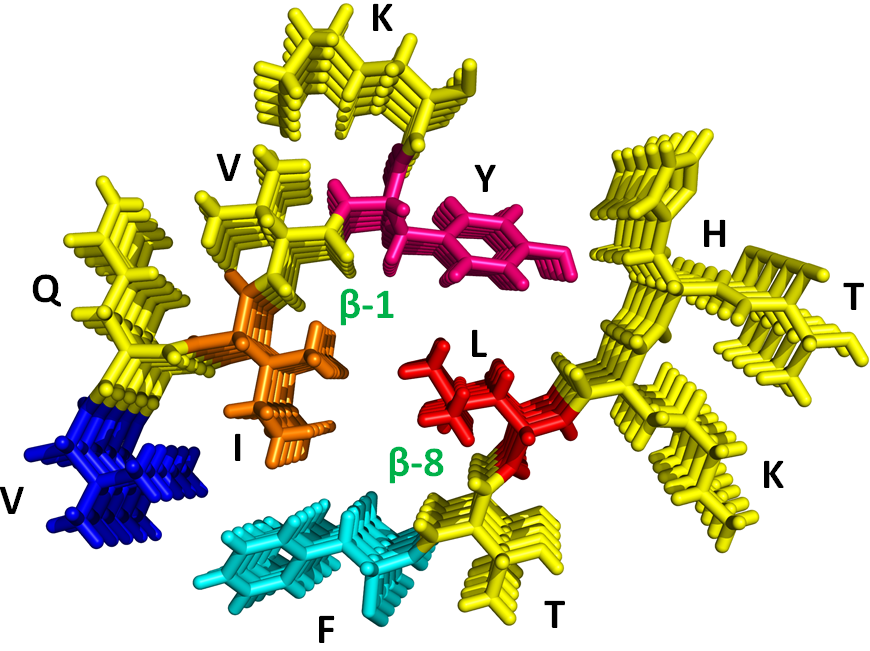


**Supplementary Figure 7**. Representation of face-to-face packing of β-1 (^306^VQIVYK^311^) and part of β-8 (^373^THKLTF^378^) involved in the β-sheet structure of tau core filament.

**Supplementary Table 1.** Description of simulated systems


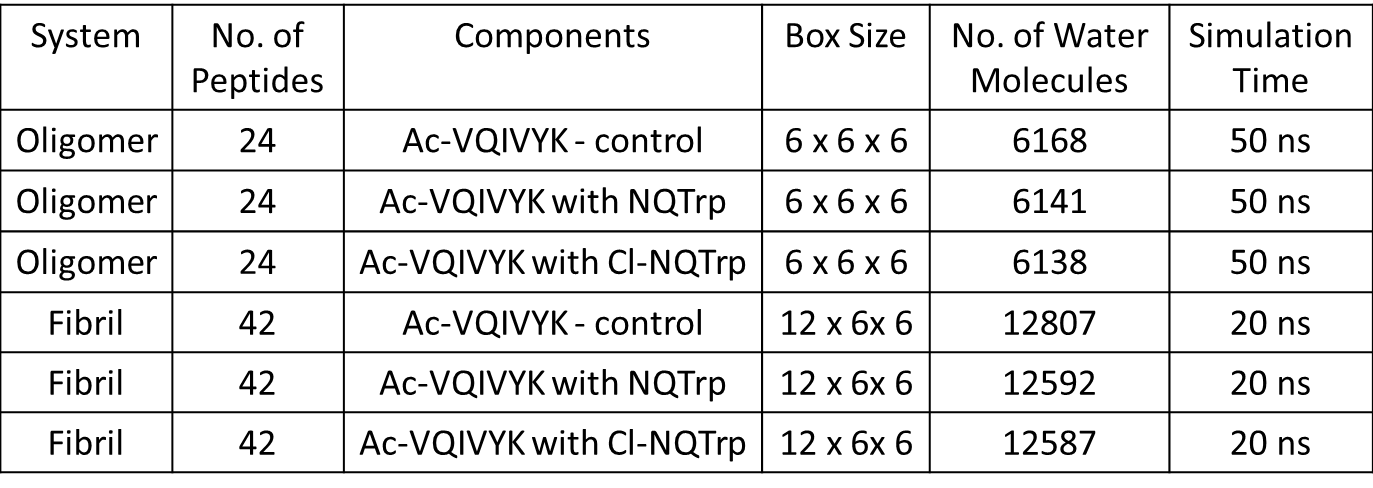

Supplement: Supplementary file 1 — Supplementary information [file 41598_2017_18443_MOESM1_ESM.doc]
